# Physical activity and prospective associations with indicators of health and development in children aged <5 years: a systematic review

**DOI:** 10.1186/s12966-020-01072-w

**Published:** 2021-01-07

**Authors:** Sanne L. C. Veldman, Mai J. M. Chin A Paw, Teatske M. Altenburg

**Affiliations:** Department of Public and Occupational Health, Amsterdam Public Health Research Institute, Amsterdam University Medical Center, Amsterdam UMC, Vrije Universiteit Amsterdam, Amsterdam, The Netherlands

**Keywords:** Physical activity, Body composition, Cardiometabolic health, Bone health, Motor development, Cognitive development, Social-emotional development, Early childhood

## Abstract

**Background:**

Early childhood is a critical period for growth and development, yet the association with physical activity during this important period is unknown. The aim of this review is to critically summarize the evidence on the prospective associations between physical activity and health and development in children aged < 5 years.

**Methods:**

A systematic search in three electronic databases (Pubmed, PsycINFO, and Sportdiscus) was conducted to identify prospective studies examining the associations between physical activity (all types; specified by quantity) and health indicators (body composition, cardiometabolic health, bone health and risks/harm) or development (motor, cognitive and social-emotional development) in young children (mean age < 5 years at baseline). Two independent researchers assessed the methodological quality using the ‘Quality Assessment Tool for Quantitative Studies’ (EPHPP). This tool covers eight quality criteria: selection bias, study design, confounders, blinding, data collection methods, withdrawals and drop-outs, intervention integrity and data-analysis.

**Results:**

Thirty-nine studies, predominantly conducted in preschoolers (ages 3–5 years), were included of which nine were rated as high methodological quality. There was moderate evidence for a positive association between physical activity and motor (*n* = 11 studies) and cognitive development (*n* = 10 studies) based on consistent findings from studies having low-to-moderate methodological quality. There was insufficient evidence for an association between physical activity and body composition (*n* = 15 studies), cardiometabolic health indicators (*n* = 7 studies), social-emotional development (*n* = 2 studies) and bone health (*n* = 2 studies) based on inconsistent findings from studies having weak-to-high methodological quality.

**Conclusions:**

There is a need for more high-quality research in order to determine the dose-response relationship between physical activity and health and development in early childhood. Special attention should be paid to studies in children below the age of 3 years.

## Background

The beneficial impact of physical activity on physical, social and cognitive health indicators is well-known in school-aged children [1, 2]. Dose-response relationships indicate the more physical activity the larger the health benefits, and at least moderate intensity physical activity is needed for substantial health benefits [2]. Interestingly, less is known about the association of physical activity with health indicators or development in children below the age of five. At this young age, children go through a critical period of growth and development as their brain develops rapidly [3]. It is therefore of great importance to determine the optimal dose of physical activity for this age group to enhance health and development. Previous reviews on the association of physical activity with health and development in early childhood [4–6] have led to different conclusions, due to a combination of different inclusion criteria (e.g. regarding study design or outcome measure) and approach for the evidence synthesis (e.g. considering the methodological quality of studies). None of the previous reviews have summarized evidence regarding the optimal dose of physical activity for this age group.

The reviews by Timmons et al. (2012) and Carson at al. (2017) both included health indicators (e.g. adiposity and cardio-metabolic health) and developmental (e.g. motor and cognitive development) outcome measures, focused on children with a mean age below 5 years and included English as well as French publications [4, 5]. Timmons et al. only included prospective designs and reported results per age group (infants: 0–1 years, toddlers: 1–3 years and preschoolers: 3–5 years) whereas the review by Carson et al. included both prospective and cross-sectional study designs and reported results per design and overall. Both reviews assessed the quality of the included studies using the Grading of Recommendations Assessment, Development, and Evaluation (GRADE) framework [4, 5, 7], scoring the criteria: risk of bias (using the Cochran risk of bias assessment [8]), inconsistency, indirectness, imprecision and other (e.g., dose-response evidence). The quality of evidence was downgraded following limitations associated with these criteria. An important notion here is that in the review of Timmons et al., a subjective measure of physical activity (e.g. parent-report) did not influence the study’s quality if this was the only weak item [4] whereas in the review of Carson et al., the use of a convenience sample and performance bias did not result in the downgrading of evidence [5]. Despite differences in reporting of outcomes (e.g. per age group or per study design), both reviews reported positive associations between physical activity and motor development, cognitive development, psychosocial health, bone and skeletal health and cardio metabolic health for one or more age groups or research designs. Timmons et al. also reported a positive association between physical activity and adiposity [4], whereas Carson et al. reported mixed findings for this association [5].

Pate et al. (2019) recently reviewed the evidence on the prospective association between physical activity and health outcomes in children up to the age of 6 years [6]. Risk of bias was assessed using the USDA Nutrition Evidence Library Bias Assessment Tool for original research [9, 10], with all items equally contributing to the overall quality scoring. Pate et al. concluded physical activity was beneficial for adiposity and bone health [6]. These results are similar to Timmons et al. [4] and partly in line with results by Carson et al. (only for bone health) [5]. In contrast to the other reviews, evidence for an association between physical activity and cardio metabolic health was insufficient and they did not consider outcomes related to children’s development [6]. For all reviews, most included studies were conducted in preschoolers (children aged 3–5 years).

Specifying the actual physical activity dose or contrast in exposure versus the reference group is essential for concluding on the association of physical activity with health and development. Therefore, and in contrast with previous reviews, the current review will apply strict inclusion criteria regarding physical activity dose (e.g. specified quantity). Additionally, the reviews described above have applied different approaches to review the literature. As such, and in combination with an increased interest in early childhood over recent years, there is a need to review the current evidence on the association of physical activity with health indicators and development in young children. The aim of this article is to critically summarize the evidence on the prospective association of physical activity with health indicators and development in children aged < 5 years. When possible, the optimal dose of physical activity will be explored by conducting a meta-analysis.

## Methods

### Protocol and registration

This systematic review was registered with the International Prospective Register of Systematic Reviews (PROSPERO), registration number: CRD42019144677. The review followed the guidelines of the Preferred Reporting Items of Systematic Reviews and Meta-analysis (PRISMA) statement [11].

### Eligibility criteria

Studies were included if they met the following criteria: 1) prospective study design (observational cohort or experimental study); 2) physical activity (all types included e.g. prone position in infants and outside play in toddlers) was assessed or clearly described (in case of experimental study) by quantity in apparently healthy children during early childhood (mean age at baseline < 5 years) and examined a prospective association with at least one of the following health indicators and developmental outcomes: motor development (e.g. gross or fine motor skills), cognitive development (e.g. executive functions, language development, concentration), social-emotional development (e.g. self-efficacy, stress, hyperactivity/impulsivity), body composition (e.g. overweight, body mass index [BMI], %body fat), growth (e.g. head circumference), bone health (e.g. bone mineral density), cardio metabolic health (e.g. fitness, blood pressure) and risks/harms (e.g. injury). For experimental studies, a difference in amount of physical activity between intervention and control group needed to be clearly described or measured; 3) article was published in English, in a peer-reviewed journal.

### Literature search and study selection

Systematic literature searches were carried out in three electronic databases: PubMed, SportDiscus (Ebsco) and PsychINFO. The search strategy focused on terms referring to study design, population, exposure and outcome measures which were linked by AND combinations. Additional file 1 provides the search strategy. Terms related to physical activity, sedentary behavior and sleep were all included as exposure. For this review, only results regarding physical activity are presented. An updated search, using only the physical activity search terms as exposure was completed on November 21st, 2019.

After removal of duplicates, one reviewer (SV) screened all titles and abstracts and 30% was independently screened by a second reviewer (RH and TA). In case of doubt, studies were included at this stage. For the updated search, all titles and abstract were screened by two reviewers (SV and TA). Full texts were independently screened by two researchers (SV and TA) to determine whether inclusion criteria were met. A third reviewer (MC) was consulted in case of inconsistencies. If a decision could not be made due to missing information (including no access to a full text article), the authors were contacted by email. Reference lists of included studies were scanned for additional relevant studies.

### Data extraction

The following data were extracted using a structured form: study methodology (e.g. design, study duration, points of data collection), participants (e.g. sample size, mean age, percentage girls), exposure (e.g. type and amount of physical activity, measurement), outcomes (e.g. outcome measure, measurement) and results. One reviewer (SV) extracted data of all included studies. A second reviewer (TA) independently extracted data of 25% of the included studies and checked the extracted data of the remaining studies. Discrepancies after the 25% data extraction by two independent reviewers was discussed until consensus was reached before the other 75% of the data extraction was performed and checked.

### Quality assessment

Two researchers (SV and TA) independently rated the methodological quality of all included studies using an adjusted version of the ‘Quality Assessment Tool for Quantitative Studies’ (EPHPP) [12, 13] (see Additional file 2). This tool contains 19 items divided over eight quality criteria: selection bias, study design, confounders, blinding, data collection methods, withdrawals and drop-outs, intervention integrity and analysis. The quality criteria blinding and intervention integrity were only applied to intervention studies. Per quality criterion, a quality score was assessed: good, fair or poor. Discrepancies were discussed until consensus was reached. The overall methodological quality of a study was classified as ‘high’ when at most one quality criteria was rated as poor and two as fair. A study was classified as ‘moderate’ when at most two quality criteria were rated as poor. The overall methodological quality of a study was classified as ‘weak’ when more than two quality criteria were rated as poor.

### Synthesis of evidence

A best evidence synthesis was applied for each of the health and developmental outcomes to draw conclusions on the level of evidence for a prospective association between physical activity and health indicators and development in children aged < 5 years. This synthesis was based on the number of studies, their methodological quality and the consistency of findings [14, 15]:

- Strong evidence: consistent findings in multiple studies (≥2) of high methodological quality.
- Moderate evidence: consistent findings in one study of high methodological quality and at least one study of weak or moderate methodological quality or consistent findings in multiple studies (≥2) of weak or moderate methodological quality.
- Insufficient evidence: only one study available, or inconsistent findings in multiple studies (≥2).
- No evidence: consistent findings for the absence of an association in multiple studies (≥2) of moderate or high methodological quality.

Results were considered consistent when ≥75% of studies demonstrated findings in the same direction, which was defined by a significance of *p <* 0.05 of the fully adjusted model. If studies examined multiple associations for the same health indictor or developmental outcome (e.g. analyzing multiple outcome measures for one health indicator), they were considered to add evidence when consistently demonstrating an association (consistent findings in ≥75% of examined associations). If two or more studies of high methodological quality were available, results of studies with weak methodological quality were ignored in determining the level of evidence.

### Meta-analyses

For each of the health and developmental outcomes it was checked whether studies were homogenous in terms of measurement of physical activity, health and developmental outcome, statistical analyses and reported types of effect sizes. As included studies varied to a large extent on these aspects, it was not possible to pool the studies examining the same health or developmental outcomes and conduct a meta-analysis.

## Results

### Study selection

**Fig. 1:**
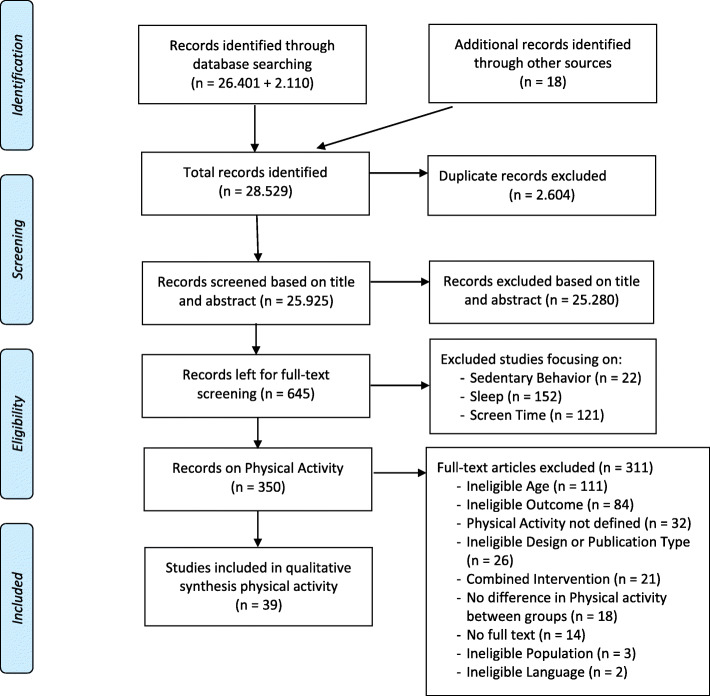
Flow Diagram for the identification, screening, eligibility and inclusion of studies

Figure 1 presents the flow diagram of included studies. The initial search identified 26,401 hits and the updated search identified 2110 hits. After removing duplicates (*n* = 2604) and checking eligibility, 21 relevant studies were eligible for inclusion. An additional 18 studies were included by scanning reference lists of included studies, resulting in a total of 39 eligible studies.

### Study characteristics

The 39 studies included 15,537 participants across 15 countries. Eighteen studies had a longitudinal design [16–33] and 21 studies an experimental design [34–54] of which 11 were randomized [34–36, 38–41, 43, 44, 46, 47]. The study duration varied between 12 months and 8 years for longitudinal studies and between 18 days and 24 months for experimental studies. Additionally, three studies examined ‘acute’ intervention effects [41, 48, 49]. Sample sizes varied between 16 and 4253 children and the percentage of girls between 23 and 69%. Six studies were conducted in children younger than 12 months [24, 25, 31, 38, 43, 54], two studies in children between one and 3 years [19, 20], and 31 studies in children between three and 5 years [16–18, 21–23, 26–30, 32–37, 39–42, 44–53]. In 46% of the studies, physical activity was assessed using an objective measurement instrument (e.g. by accelerometer or heart rate monitor). Six different health indicators and development outcomes were examined, with body composition (*n* = 15 studies [30]) [16–23, 31, 33, 34, 37, 40, 42], motor development (*n* = 11 studies) [25, 35–38, 42, 43, 45, 52–54] and cognitive development (*n* = 10 studies) [24, 39, 41, 44, 46–51] most frequently reported. Seven studies examined more than one indicator of health and development [16, 24, 30, 33, 36, 37, 42]. Additional file 3 includes Tables S1-S6 that display details on study design, sample, exposure, outcome, and main findings for all included studies.

### Quality assessment

**Fig. 2:**
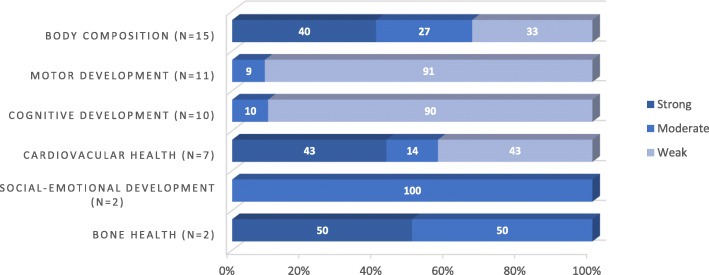
Summary of methodological quality across reviewed studies per outcome

Nine out of 39 studies were rated as high methodological quality [18, 20, 21, 23, 27, 28, 31–33], eight studies were rated as moderate quality [16, 17, 19, 22, 24–26, 29] and 22 studies were rated as weak methodological quality [30, 34–54]. Figure 2 displays a summary of the methodological quality across included studies per outcome. Overall, the item ‘selection bias’ received the lowest score (item A; see Table S7 in Additional file 4). For intervention studies, the items ‘intervention integrity’ and ‘blinding’ (items D and G) received the lowest scores.

### Data synthesis

A summary of results per health- and developmental outcome is displayed in Table 1.

**Table 1:** Summary of results

Health or developmental outcome	Intervention studies^**a**^	Longitudinal studies^**a**^	Total effect and evidence
**Body composition** *(15 studies, 3448 participants)*^*a*^			
BMI	0 0 0	+ **0 0 0 0** 0 **-** - -	Based on inconsistent findings among the studies with high methodological quality, there is insufficient evidence for the effects of physical activity on body composition in young children (< 5 yrs).
Fat percentage	+ 0 0	**+** 0	
Fat mass	+	**+** + **0 0** 0 0	
Fat free mass	0	+ + **0**	
Waist/hip ratio		+	
Weight		0 -	
**Motor development** *(11 studies, 1281 participants)*			
Total motor score	+ + + + 0	0	Based on consistent findings among studies with weak-to-moderate methodological quality, there is moderate evidence for the positive effects of physical activity on motor development in young children (< 5 yrs).
Total gross motor score	0		
Total fine motor score		0	
Locomotor score	+ +		
Object control score	+ + 0		
Balance score	+ +		
Manual dexterity score	0		
Gross motor milestones	+	+	
**Cognitive development** *(10 studies, 4939 participants)*			
School outcomes (e.g. math, language)	+ + + + + +	–	Based on consistent findings among studies with weak-to-moderate methodological quality, there is moderate evidence for the positive effects of physical activity on cognitive development in young children (< 5 yrs).
Acute effects (e.g. concentration)	+ + 0		
**Cardiovascular health** *(7 studies, 1381 participants)*			
Blood pressure	+	**+ 0**	Based on inconsistent findings among the studies with high methodological quality, there is insufficient evidence for the effects of physical activity on cardiovascular health in young children (< 5 yrs).
Insulin		**0 0**	
Triglycerides		**+** 0	
Cholesterol		**0** 0	
Adiponectin		**+**	
Glucose, leptin, hsCRP		**0**	
Lipoprotein		0	
Total metabolic score		**+**	
Serum lipids and lipoproteins		0	
Cardiovascular fitness		**+** +	
Arterial stiffness		+	
**Social-emotional development** *(2 studies, 7038 participants)*			
Behavior		–	Based on inconsistent findings and a limited amount of available studies, there is insufficient evidence for the effects of physical activity on social-emotional development in young children (< 5 yrs).
Quality of Life		0	
**Bone health** *(2 studies, 2907 participants)*			
Bone density		**0**	Based on inconsistent findings and a limited amount of available studies, there is insufficient evidence for the effects of physical activity on bone health in young children (< 5 yrs).
Fractures		–	

^a^Only studies that conducted statistical analyses are included in the summary table; + = study adding evidence for a positive association; − = study adding evidence for a negative association; 0 = study indicating no evidence for an association; ^a^ = a positive association means positive for children’s health; **Bold** values = studies of high methodological quality; *BMI* Body mass index, *hsCRP* High-sensitivity C-reactive protein

#### Body composition

Fifteen studies examined the association between physical activity and body composition [16–23, 30, 31, 33, 34, 37, 40, 42], of which four studies were intervention studies [16–23, 30, 31, 33] (See Table 1 and Table S1). Body mass index (BMI) was the most commonly examined outcome measure (*n* = 13 studies) [16–23, 33, 34, 37, 40, 42], followed by fat mass (*n* = 7 studies) [16, 17, 22, 23, 31, 33, 34], fat percentage (*n* = 6 studies) [20, 22, 34, 37, 40, 42], fat-free mass (*n* = 4 studies) [16, 22, 23, 34], weight (*n* = 2 studies) [19, 22] and waist/hip ratio (*n* = 1 study) [30]. Nine studies measured physical activity objectively (e.g. by accelerometer) [16, 17, 19, 21–23, 31, 33, 34] and six studies subjectively (e.g. by parent-report) [18, 20, 30, 37, 40, 42]. Six of the 15 included studies were rated as high quality [18, 20, 21, 23, 31, 33], four studies as moderate quality [16, 17, 19, 22] and five studies as weak quality [30, 34, 37, 40, 42].

Five of six high quality studies examined the association between physical activity and BMI [18, 20, 21, 23, 33]. Four studies found no significant association between physical activity and BMI [18, 20, 23, 33] whereas one study found a positive association [21]. Three studies examined the association between physical activity and fat mass, of which one study found a negative association [31] and two studies found no significant association [23, 33]. One study examined the association between physical activity and fat percentage and found a negative association for girls but not for boys [20]. No significant association was found between physical activity and fat free mass [23].

Based on inconsistent findings among the studies with high methodological quality, there is insufficient evidence for an association between physical activity and body composition in children under the age of 5 years.

#### Motor development

Eleven studies examined the association between physical activity and motor development outcomes [25, 35–38, 42, 43, 45, 52–54] (See Table 1 and Table S2). Outcome measures included total motor scores (gross motor, fine motor or a combination; *n* = 7 studies) [25, 37, 38, 43, 45, 53, 54], a specific component of gross or fine motor skills (e.g. ball skills; *n* = 5 studies) [35, 38, 52–54] and individual motor skills (e.g. jump, *n* = 8 studies) [25, 35, 36, 38, 42, 43, 53, 54]. Ten studies were intervention studies [35–38, 42, 43, 45, 52–54] with a large variety in frequency of implemented physical activity sessions (one session per week to daily), session duration (15 to 60 min per session), study duration (3 weeks to 2 years) and intervention content (e.g. time in prone position in infants, movement program in preschoolers). Physical activity was measured subjectively in all studies. One of the eleven studies was rated as moderate quality [25] whereas ten studies were rated as weak quality [35–38, 42, 43, 45, 52–54].

All intervention studies found a positive association between physical activity and motor development (either total score, a specific component or an individual skill). Two of these studies only found an association for separate skills or components but not for the total (gross) motor score [53, 54] and one study did not perform statistical analyses [42]. The longitudinal study found a positive association between prone experience and duration and the attainment of motor milestones [25].

Based on consistent findings among studies with weak-to-moderate methodological quality, there is moderate evidence for a positive association of physical activity with motor development in children under the age of 5 years.

#### Cognitive development

Ten studies examined the association between physical activity and cognitive outcomes of which nine studies were intervention studies [39, 41, 44, 46–51] (See Table 1 and Table S3). Seven studies examined associations with school-related outcomes such as language and math [24, 39, 44, 46, 47, 50, 51] and three studies examined acute effects on attention, concentration and/or response inhibition [41, 48, 49]. Physical activity was measured both objectively (e.g. accelerometer; *n* = 6 studies) [39, 41, 44, 46, 47, 49] and subjectively (*n* = 4 studies) [24, 48, 50, 51]. One study was rated as moderate quality [24] whereas nine studies were rated as weak quality [39, 41, 44, 46–51].

Four intervention studies, lasting between one and 4 weeks, examined the effects of physical activity on learning outcomes [39, 44, 46, 47], randomly assigning children to: 1) an integrated physical activity condition including task-relevant physical activity, 2) a non-integrated physical activity condition involving task-irrelevant physical activity, 3) a control condition without physical activity. All four studies showed that children in the task-related physical activity group scored best on learning outcomes [39, 44, 46, 47]. Two studies, lasting six and 8 months respectively, examined the association of physically active academic lessons on early literacy and language in comparison to a regular academic lesson control group and showed positive intervention effects [50, 51]. Three intervention studies examined the acute effects of physical activity (range 10–30 min) on attention, concentration and/or response inhibition [41, 48, 49]. The intensity was described as moderate-to-vigorous intensity physical activity in two studies [41, 49] while in the third study the intervention was described as ‘recess time’ without specified intensity [48]. Results on acute effects of physical activity were mixed. One study showed a positive effect of 30-min physical activity on classroom attention [47], one study did not find an effect of two 10-min physical activity breaks on concentration [53] and one study showed a positive effect of 20-min physical activity recess compared to 10- or 30-min recess on attention and concentration [48].

Based on consistent findings among studies with weak-to-moderate methodological quality, there is moderate evidence for a positive association of physical activity with cognitive development in children under the age of 5 years.

#### Cardiometabolic health indicators

Seven studies examined the association between physical activity and cardiometabolic health indicators such as blood pressure [27, 33, 36, 42], biomarkers [30, 32, 33] and physical fitness [16, 27], of which two studies were intervention studies [36, 42] (See Table 1 and Table S4). Physical activity was measured objectively in four studies [16, 27, 32, 33] and subjectively in three studies [30, 36, 42]. Three studies were rated as high methodological quality [27, 32, 33], one study was rated as moderate methodological quality [16] and three studies were rated as weak methodological quality [30, 36, 42].

Of the three studies rated as high quality, two studies examined the association between physical activity and blood pressure [27, 33]. One study found mixed results (positive association in boys but not in girls) [33] whereas the other study found no significant association between physical activity and blood pressure [27]. Two studies found some positive associations when examining the association between physical activity and biomarkers, i.e. adiponectin [32], metabolic z-scores [33] and triglycerides [33]. A positive association between physical activity and physical fitness was found in one study [27].

Based on inconsistent findings among the studies with high methodological quality, there is insufficient evidence for associations of physical activity with cardiometabolic health indicators in children under the age of 5 years.

#### Social-emotional development

Two studies examined the association between physical activity and social-emotional development [24, 26] (See Table 1 and Table S5). Both studies had a longitudinal design, measured physical activity subjectively and were rated as moderate methodological quality [24, 26]. One study observed an increase in externalizing behavior with increasing physical activity [24], whereas the other study did not find an association between physical activity and quality of life [26].

Based on inconsistent findings in two studies of moderate methodological quality, there is insufficient evidence for an association of physical activity with social-emotional development in children under the age of 5 years.

#### Bone health

Two studies examined the association between physical activity and bone health: fractures [29] and bone density [28] (See Table 1 and Table S6). Both studies had a longitudinal design [28, 29] and physical activity was measured objectively in one study [28]. The methodological quality was rated as high [28] and moderate quality [29]. No significant association between physical activity and bone density was found [28] while time spent in outdoor play in summer was associated with an increased risk of fractures [29].

Based on inconsistent findings in two studies of moderate-to-high methodological quality, there is insufficient evidence for an association of physical activity with bone health in children under the age of 5 years.

## Discussion

This systematic review summarized the evidence on the prospective associations of physical activity with health indicators and development in children aged < 5 years. We found moderate evidence for a positive association of physical activity with motor development and cognitive development. Although associations were consistently positive, the methodological quality of most of the included studies was weak. For other outcomes, such as body composition, cardiometabolic health indicators, social-emotional development and bone health, the evidence was insufficient due to inconsistent findings.

Conclusions from the current review were partly in line with previous reviews. Results relating to motor and cognitive development were comparable, but in contrast to previous reviews, the current review found no evidence for an association between physical activity and body composition [4, 6], bone health [4–6], cardiometabolic health [4, 5] or social-emotional development [4, 5]. One explanation might be the difference in inclusion criteria. Compared to the other systematic reviews, we applied stricter inclusion criteria especially regarding the exposure variable physical activity. Studies were only included if the amount of physical activity was quantified and for experimental studies, a difference in amount of physical activity between intervention and control group needed to be clearly described or measured. In many studies it was not clear whether children in the intervention group were exposed to more physical activity than children in the control group, which led to exclusion. In terms of methodological quality assessment, all reviews assessed similar quality items but two reviews excluded some items (e.g. no downgrading of evidence for a subjective measure of physical activity, the use of a convenience sample or performance bias) from the overall quality score [4, 5]. In the present review, we included all quality items in the overall methodological quality score and applied an evidence synthesis to combine number of studies, methodological quality and consistency of findings. This, in combination with the difference in included studies, resulted in somewhat different conclusions.

An important methodological limitation is that more than half of the studies did not used valid and reliable measures of physical activity. One reason is the absence of valid and reliable physical activity assessment tools for this young age group, especially in children under the age of 2 years. This might explain the low number of available studies exploring the association of physical activity with health indicators and development in children under the age of 3 years. In addition, a large variation in physical activity was reported (e.g. time spent in moderate-to-vigorous physical activity, physical education or aerobic dance). This prohibited drawing conclusions regarding the optimal type, intensity, frequency and duration of physical activity for health and developmental benefits. Other common methodological issues identified in this review were studies not including a representative sample and the lack of reporting on recruitment rates.

Body composition was the most frequently examined outcome, which is in line with previous reviews [4–6]. One explanation for insufficient evidence for an association between physical activity and body composition may be the lack of adjustment for diet. Diet is an important component of bodyweight and as such should be adjusted for in analysis when examining the association between physical activity and body weight. As diet was not adjusted for in every study, this could explain the difference in results found and therefore the conclusion insufficient evidence. Furthermore, inaccurate measures of physical activity can explain the results.

We found moderate evidence for a positive association of physical activity with motor development, which is in line with several previously published reviews [4, 5, 55]. An important note is that most of the included studies on motor development were intervention studies. As such, the positive association between physical activity and motor development in the current review may be attributed to the instruction on the quality of movement (e.g. motor skill instruction during interventions) rather than the amount of physical activity itself. When examining the literature on motor skills interventions, the effectiveness of these interventions at improving motor skills through motor skill instruction has been demonstrated at several occasions [56–58].

Results from the current review confirm the previous evidence on the association of physical activity with cognitive development in children [5, 55, 59, 60]. However, included studies varied strongly in the amount of physical activity, the study duration, the cognitive outcomes examined and the measurement tools used. Additionally, there is a lack of studies of high methodological quality. As such, more high-quality research is needed to confirm this association, both potential acute as well as long-term effects, that allows for dose-response analysis.

### Strengths and limitations

The strength of this review was the inclusion of studies with a prospective design and the strict inclusion criteria regarding physical activity volume. Additional strengths include the thorough and extensive literature search, the contacting of authors in case of missing information, the large number of outcome variables included in the search, the methodological quality assessment and the best evidence synthesis. The main limitation of this review is the heterogeneity of included studies examining the same health or developmental outcome making statistical pooling of included studies inappropriate. Therefore, we could not conduct dose-response meta-analyses. Furthermore, we could not evaluate potential publication bias or selective reporting of significant findings, which is a limitation of our review. Another limitation is only including publications in English.

### Recommendations for future research

To increase the evidence-base for physical activity guidelines for the early years, we have the following recommendations for future research:

1. Conducting high quality randomized controlled trials and prospective cohort studies to examine the dose-response relationships between physical activity and health indicators and developmental outcomes such as body composition, bone and skeletal health, cardiometabolic health, motor development, cognitive development and social-emotional development, especially for children under the age of 3 years. Additionally, to enable future meta-analyses, we urge for consensus on outcome measures (preferably developing a core outcome set [61]) including preferred valid and reliable assessment tools;
2. Develop and validate methods for accurate measurement of physical activity in the early years, especially for children under the age of 2 years; and
3. Improve the quality of reporting studies especially regarding recruitment, blinding of outcome assessors and intervention integrity such as intervention delivery, consistency and potential contamination between intervention and control group.

## Conclusion

This systematic review examined the evidence on the prospective association of physical activity with health indicators and development in children aged < 5 years. We found moderate evidence for a positive association of physical activity with motor and cognitive development. We found insufficient evidence for an association of physical activity with body composition, cardio-metabolic health indicators, social-emotional development and bone health. More high-quality research is needed to identify optimal types, intensity, frequency and duration of physical activity for health and developmental that can inform physical activity guideline development for the early years. Special attention should be given to children below the age of 3 years, as in this young age group only few studies are available.

## Supplementary Information

**Additional file 1.** Overview of search terms – This additional file contains the search terms used to conduct the search across three electronic databases: PubMed, SportDiscus (Ebsco) and PsychINFO. The search strategy focused on terms referring to study design, population, exposure and outcome measures which were linked by AND combinations.

**Additional file 2.** Quality assessment tool for quantitative studies – This additional file contains the adjusted version of the ‘Quality Assessment Tool for Quantitative Studies’ (EPHPP). This tool was used to assess the methodological quality of the included studies.

**Additional file 3: Tables S1-6.** This additional file includes six tables that display details on study design, sample, exposure, outcome, and main findings for all included studies (Tables S1-6, one table per outcome measure).

**Additional file 4: Table S7.** This additional file includes Table S7 that displays the methodological quality of all included studies.

## Data Availability

Not applicable.
