# Supplementary material for: Physical activity and prospective associations with indicators of health and development in children aged <5 years: a systematic review
Source: Int J Behav Nutr Phys Act. 2021 Jan 7;18:6. doi: 10.1186/s12966-020-01072-w (PMC7791660; doi:10.1186/s12966-020-01072-w)
Supplement: Supplementary file 1 — Additional file 1. Overview of search terms – This additional file contains the search terms used to conduct the search across three electronic databases: PubMed, SportDiscus (Ebsco) and PsychINFO. The search strategy focused on terms referring to study design, population, exposure and outcome measures which were linked by AND combinations. [file 12966_2020_1072_MOESM1_ESM.docx]

**Additional File 1. Overview of search terms**

Initial search, conducted December 20th 2018

((((((cohort OR longitudinal OR randomized controlled trial OR prospective OR follow up)) AND (child OR pediatr* OR boy* OR girl* OR infant* OR baby OR babies OR toddler* OR preschool* OR childcare OR daycare OR early childhood)) AND (physical activit* OR exercise* OR sport* OR active* OR walk* OR run* OR aerobic OR outdoor OR play* OR bicycle* OR biking OR tummy time OR floor time OR prone position OR crawl* OR swim* OR rough and tumble OR sedentary OR sitting OR physical* inactive* OR computer time OR computer use OR screen time OR sedentary activity OR sedentary activities OR television OR videogame OR gaming OR tablet use OR tablet time OR screen entertainment OR screen based OR sleep OR nap)) AND (health* OR social behav* OR prosocial behav* OR behavio* conduct OR conduct disorder* OR aggression OR cognitive dev* OR academic achievement OR language dev* OR verbal OR vocabulary OR self control OR self regulation OR self efficacy OR executive function OR memory OR concentrate* OR cognition OR cognitive* OR cognitive processes OR cognitive ability OR learn* OR neuropsychological test OR attention* OR attention control OR attention span OR verbal development OR spatial ability OR perception OR intelligence OR speech OR academic achievement OR psychomotor performance OR motor development OR motor skills OR motor activit* OR movement skills OR physical competenc* OR physical* litera* OR gross motor OR motor performance OR object manipulation OR motor coordination OR actual competence OR object control OR locomotor skills OR motor proficiency OR motor competence OR balance OR stability OR postural balance OR psychosocial OR psycho social OR interpersonal OR depression OR depressive OR depressed OR mood disorder* OR anxiety OR distress OR worry OR psychological stress* OR self esteem OR self concept OR bone density OR bone mass OR absorptiometry* OR densitometry* OR photodensitometry OR dxa OR dexa OR hypertens* OR high blood pressure OR cholesterol OR hypercholester* OR hyper lipid* OR dyslipid* OR lipids* OR lipoprotein* OR head circumference OR obese OR obesity OR overweight OR body mass index OR bmi OR waist OR adipose* OR fat OR body composition OR skin fold* OR skinfold* OR metabolic syndrome OR insulin resistance OR diabet* OR glucose OR fitness OR physical condition* OR cardiovascular OR child develop* OR developmental milestone* OR injury*))) NOT (cross-sectional)

Updated search, conducted November 21^st^, 2019

((((cohort OR longitudinal OR "randomized controlled trial" OR prospective OR "follow up")) AND (child OR boy* OR girl* OR infant* OR baby OR babies OR toddler* OR preschool* OR childcare OR daycare OR "early childhood")) AND (physical activit* OR exercise* OR sport* OR active* OR walk* OR run* OR aerobic OR outdoor OR play* OR bicycle* OR biking OR "tummy time" OR "floor time" OR "prone position" OR crawl* OR swim* OR "rough and tumble")) AND (health* OR social behav* OR prosocial behav* OR behavio* conduct OR conduct disorder* OR aggression OR cognitive dev* OR "academic achievement" OR language dev* OR verbal OR vocabulary OR "self control" OR "self regulation" OR "self efficacy" OR "executive function" OR memory OR concentrate* OR cognition OR cognitive* OR "cognitive processes" OR "cognitive ability" OR learn* OR "neuropsychological test" OR attention* OR "attention control" OR "attention span" OR "verbal development" OR "spatial ability" OR perception OR intelligence OR speech OR "academic achievement" OR "psychomotor performance" OR "motor development" OR "motor skills" OR motor activit* OR "movement skills" OR physical competenc* OR physical* litera* OR "gross motor" OR "motor performance" OR "object manipulation" OR "motor coordination" OR "actual competence" OR "object control" OR "locomotor skills" OR "motor proficiency" OR "motor competence" OR balance OR stability OR "postural balance" OR psychosocial OR "psycho social" OR interpersonal OR depression OR depressive OR depressed OR mood disorder* OR anxiety OR distress OR worry OR psychological stress* OR self esteem OR self concept OR bone density OR bone mass OR absorptiometry* OR densitometry* OR photodensitometry OR dxa OR dexa OR hypertens* OR "high blood pressure" OR cholesterol OR hypercholester* OR hyper lipid* OR dyslipid* OR lipids* OR lipoprotein* OR "head circumference" OR obese OR obesity OR overweight OR "body mass index" OR bmi OR waist OR adipose* OR fat OR "body composition" OR skin fold* OR skinfold* OR "metabolic syndrome" OR "insulin resistance" OR diabet* OR glucose OR fitness OR physical condition* OR cardiovascular OR child develop* OR developmental milestone* OR injury*) NOT cross-sectional [tiab]

Filters: Publication date from 2018/12/20
