# Supplementary material for: Physical activity and prospective associations with indicators of health and development in children aged <5 years: a systematic review
Source: Int J Behav Nutr Phys Act. 2021 Jan 7;18:6. doi: 10.1186/s12966-020-01072-w (PMC7791660; doi:10.1186/s12966-020-01072-w)
Supplement: Supplementary file 2 — Additional file 2. Quality assessment tool for quantitative studies – This additional file contains the adjusted version of the ‘Quality Assessment Tool for Quantitative Studies’ (EPHPP). This tool was used to assess the methodological quality of the included studies. [file 12966_2020_1072_MOESM2_ESM.docx]

**Additional File 2. Quality Assessment Tool for Quantitative Studies’ (EPHPP)** [1, 2]**.**

**A. SELECTION BIAS**

| Q1 Representative | Very likely^1^ | Very/Somewhat likely^2^ | Not likely^3^/Not described |
| --- | --- | --- | --- |
| Q2 Participation | 80-100% | 80-100%/60-79% | Less than 60%/can’t tell |
| RATING | GOOD | FAIR | POOR |

^1^ Random individuals selected from target population.
^2^ Referred systematically from schools/settings.

^3^ Self-referred

**B. STUDY DESIGN**

| Design | Longitudinal design/RCT^1^ | RCT^1^ or Controlled clinical trial | Other/can’t tell |
| --- | --- | --- | --- |
| Randomized | NA/YES | NO | No/NA |
| Described as randomized | NA/YES | NO | No/NA |
| RATING | GOOD | FAIR | Poor |

^1^ Randomized controlled trial.

**C. CONFOUNDERS**

| Controlled for confounders | Controlled for pre-intervention/baseline scores, sex and age | Controlled for pre-intervention/baseline scores | Not controlled for pre-intervention/baseline scores |
| --- | --- | --- | --- |
| RATING | GOOD | FAIR | POOR |

**D. BLINDING**

| Q1 Assessors | YES |  | NO / can’t tell | NA |
| --- | --- | --- | --- | --- |
| RATING | GOOD | FAIR | POOR | NA |

**E. DATA COLLECTION METHODS**

| Q1 Exposure: valid | Objective or validity >0.7: YES/NO/Can’t tell | At least three times a YES score | Two times a YES score | No or one YES score |
| --- | --- | --- | --- | --- |
| Q2 Exposure: reliable | Objective or reliability >0.7: YES/NO/Can’t tell |  |  |  |
| Q3 Outcome: valid | Validity >0.7: YES/NO |  |  |  |
| Q4 Outcome: reliable | Reliability >0.7: YES/NO/Can’t tell |  |  |  |
| RATING |  | GOOD | FAIR | POOR |

**F. WITHDRAWALS AND DROP-OUTS**

| Q1 Reported | YES | YES/NO | YES/NO |
| --- | --- | --- | --- |
| Q2 Completion | 80-100% | 80-100%/60-79% | <60% / can’t tell |
| RATING | GOOD | FAIR | POOR |

**G. INTERVENTION INTEGRITY**

| Q1 Intervention delivery | 80-100% | 80-100%/60-79% | <60% / can’t tell | NA |
| --- | --- | --- | --- | --- |
| Q2 Consistency measured | YES | YES | NO/Can’t tell | NA |
| Q3 Contamination between groups | NO | NO | YES/Can’t tell | NA |
| RATING | GOOD | FAIR | POOR | NA |

**H. ANALYSES**

| Q1 Allocation / analysis unit^5^ | Similar/NA | Not similar | Not similar / can’t tell |
| --- | --- | --- | --- |
| Q2 Subjects = 10x variables | YES | YES | NO/can’t tell |
| Q3 Analyses appropriate^6^ | YES | YES | NO/can’t tell |
| Q4 Intention to treat^7^ | YES/NA | YES/can’t tell/NA | NO/can’t tell/NA |
| RATING | GOOD | FAIR | POOR |

^5^ Level of allocation and analysis should be the same for a strong analysis (multi-level), otherwise weak

^6^ Effect sizes reported, i.e. beta.

^7^ Only applicable for longitudinal studies

**OVERALL METHODOLOGICAL QUALITY SCORE**

**HIGH:** MAX ONE POOR SCORE AND MAXIMUM 2 FAIR SCORES

**MODERATE:** MAX. TWO POOR SCORE

**WEAK:** >2 POOR SCORE
