# Supplementary material for: Physical activity and prospective associations with indicators of health and development in children aged <5 years: a systematic review
Source: Int J Behav Nutr Phys Act. 2021 Jan 7;18:6. doi: 10.1186/s12966-020-01072-w (PMC7791660; doi:10.1186/s12966-020-01072-w)
Supplement: Supplementary file 3 — Additional file 3: Tables S1-6. This additional file includes six tables that display details on study design, sample, exposure, outcome, and main findings for all included studies (Tables S1-6, one table per outcome measure). [file 12966_2020_1072_MOESM3_ESM.docx]

**Supplementary Table 1. Participant and study characteristics, main findings and methodological quality of included studies for body composition.**

| **Reference (Author, year, country)** | **Sample (size (n), age, %girls)** | **Physical activity exposure** | **Outcome measure** | **Main findings*** | **Methodological quality** |  |
| --- | --- | --- | --- | --- | --- | --- |
| **Intervention studies** | | | | | | |
| Goldfield et al. (2016), Canada [1] | n=83;  INT: n=40,  3.3±0.6yrs, 55%g;  CON: n=43,  3.3±0.6yrs, 46.7%g | INT: 60min/d teacher-led MVPA for 6mths^1^  CON: Standard curriculum  *Note: INT = 22.5min/d PA more than CON^2^* | BMI, fat mass, fat free mass and fat percentage after 6mths | Children in the intervention group had a higher decrease in fat percentage  (-1.9%, 95%CI=-3.5, -0.3) and fat mass  (-0.3kg, 95% CI=-0.7, -0.1) compared to the control group. | Weak |  |
| Krombholz et al. (2012), Germany [2] | n=428;  INT: n=211, 55.1±7.3mths, 48%g;  CON: n=217, 54.4±7.8mths, 48.4%g | INT: 1x/wk 45min teacher-led physical education + minimal 20min/d PA (for example games) for 20mths^1^  CON: 1x/wk 45min physical education | BMI and fat percentage after 20mths | No differences between groups at follow-up. | Weak |  |
| Mo-suwam et al. (1998), Thailand [3] | n=292;  INT: n=147,  4.5±0.4yrs, 44%g;  CON: n=145,  4.5±0.4yrs, 39.3%g | INT: 3x/wk 15min morning walk and 20min aerobics dance session led by trained personnel in the afternoon for 29-30wks^1^  CON: Routine PA at Kindergarten | BMI and fat percentage after 30wks | No differences between groups at follow-up. | Weak |  |
| Scheffler et al. (2007), Germany [4] | n=127, 3yrs, 55.2%g | INT: 3x/wk 60min teacher-led movement program for 24mths^1^  CON: No movement program | BMI and fat percentage after 24mths | After completion of the intervention, children in the intervention group had a fat percentage of 16.3% and BMI of 16.6kg/m^2^ and children in the control group had a fat percentage of 17.3% and BMI of 16.4kg/m^2^.  *Note: No statistical analysis conducted.* | Weak |  |

* = Only significant results, determined at p<0.05, displayed, INT = intervention group, CON = Control group, ^1^ = Reported intervention dose, ^2^ = Valid and reliable measurement/objectively measured physical activity, ^3^ = No valid and/or reliable measurement/subjectively measured physical activity, PA = Physical activity, MVPA = Moderate-to-vigorous intensity physical activity, BMI = Body mass index, n = number, %g = percentage of girls, yr(s) = year(s), mths = months, wk(s) = week(s), d = day(s), hr(s) = hour(s), min = minute(s), sec = seconds, kg = kilogram(s), m = meter.

**Supplementary Table 1. Participant and study characteristics, main findings and methodological quality of included studies for body composition (continued).**

| **Reference (Author, year, country)** | **Sample (size (n), age, %girls)** | **Physical activity exposure** | **Outcome measure** | **Main findings*** | **Methodological quality** |  |
| --- | --- | --- | --- | --- | --- | --- |
| **Longitudinal studies** | | | | | | |
| Carter et al. (2011), Nieuw Zeeland [5] | n=202, 3yrs, 43%g | Total PA over three years, per age group:  3yrs = 467±146cnts/min,  4yrs = 293±116cnts/min,  5yrs = 284±126cnts/min^2^ | BMI, fat mass and fat free mass at age 7 | No association between PA and BMI, fat mass or fat free mass. | High |  |
| De Coen et al. (2013), Belgium [6] | n=473, 4.9±1.3yrs, 48.8%g | PA at baseline:  At home = 6.0±2.6 hr/wk  Organized PA = 4.1±4.7hr/wk^3^ | BMI after 30mths | No association between PA and BMI. | High |  |
| Jago et al. (2005), USA [7] | n=133, 4.4±0.6yrs, 51%g | MVPA over three years, per year:  yr 1 = 4.2±3.6min/hr,  yr 2 = 4.2±3min/hr,  yr 3 = 3.6±3.6min/hr^2^ | BMI across 36mths | More MVPA was associated with a higher BMI (B=7.7min/hr PA, SE=2.6, β= 0.2). | High |  |
| Metcalf et al. (2008), United Kingdom [8] | n=212, 4.9yrs, 46.7%g | PA (≥3 METs) over 48mths =  g: 45min/d,  b: 57min/d^2^ | BMI and fat mass over 4yrs | No association between minutes spent above 3METs and BMI or fat mass. | High |  |
| Saldanha-Gomes et al. (2017), France [10] | n=883, 2yrs, 46%g | Outdoor play at baseline per activity level:  Low: g=38.3%/d, b=28.5%/d,  Intermediate: g=33.2%/d, b=34.1%/d,  High: g=28.5%/d, b=37.4%/d^3^ | BMI, fat percentage at age 5 | A higher level of outdoor play was associated with a lower fat percentage compared to the low activity level (high: -1%, 95%CI=-1.6, -0.3) in girls. | High |  |
| Wells & Ritz (2001), United Kingdom [9] | n=19, 0.9±0.1yrs | Total EE = 3110±640kJ/d^2^  Movement behavior (awake and active) at 9 or 12mths | Fat mass: skinfold thickness triceps and subscapular and Fat Mass Index at 24mths | Higher infant levels of movement behavior were associated with lower skinfolds thickness (β=-0.59mm, SE=0.25). | High |  |

* = Only significant results, determined at p<0.05, displayed, ^1^ = Reported intervention dose, ^2^ = Valid and reliable measurement/objectively measured physical activity, ^3^ = No valid and/or reliable measurement/subjectively measured physical activity, PA = Physical activity, MVPA = Moderate-to-vigorous intensity physical activity, EE = Energy expenditure, BMI = Body mass index, n = number, %g = percentage of girls, yr(s) = year(s), mths = months, wk(s) = week(s), d = day(s), hr(s) = hour(s), min = minute(s), sec = seconds, cnts = counts, m = meter, mm = millimeters.

**Supplementary Table 1. Participant and study characteristics, main findings and methodological quality of included studies for body composition (continued).**

| **Reference (Author, year, country)** | **Sample (size (n), age, %girls)** | **Physical activity exposure** | **Outcome measure** | **Main findings*** | **Methodological quality** |
| --- | --- | --- | --- | --- | --- |
| **Longitudinal studies** | | | | | |
| Butte et al. (2016), USA [11] | n=111, 4.6±0.9yrs, 47.7%g | PA at baseline:  Activity counts = 118.6±22.7*10^4^ cnts/d, MVPA = 54±24 min/d,  total EE = 1202±200kcal/d,  activity EE = 194±132kcal/d,  level of PA = 1.35±0.1 (range 1-5)^2^ | BMI, fat mass, fat free mass, fat percentage and weight over 12mths | MVPA was positively associated with BMI (0.01 kg/m^2^; SE=0.003) and fat free mass (0.01kg; SE=0.003).  Total EE and activity EE was positively associated with BMI (0.002 kg/m^2^; SE=0.0004), fat mass (0.001kg; SE=0.001), fat free mass (0.002kg; SE=0.001) and weight (0.003kg; SE=0.001).  PA level was positively associated with BMI (1.3 kg/m^2^; SE=0.5), fat free mass (1.3kg; SE=0.5) and weight (1.9kg; SE=0.7). | Moderate |
| Ip et al. (2016), USA [12] | n=244, 2.5-3.5yrs (53% 2yrs; 48% 3yrs), 51.6%g | Total PA over 19mths per activity profile:  ‘Less active state’:  LPA = 405.2±70 min/d,  MVPA = 6.2±3.7min/d;  ‘More active state’:  LPA = 397.3±81.7min/d,  MVPA = 19±7.8min/d)^2^ | BMI and weight percentile over 19mths | No association between the activity profile and BMI or weight percentile. | Moderate |

* = Only significant results, determined at p<0.05, displayed, ^1^ = Reported intervention dose, ^2^ = Valid and reliable measurement/objectively measured physical activity, ^3^ = No valid and/or reliable measurement/subjectively measured physical activity, PA = Physical activity, LPA = Light intensity physical activity, MPA = Moderate intensity physical activity, VPA = Vigorous intensity physical activity, MVPA = Moderate-to-vigorous intensity physical activity, EE = Energy Expenditure, BMI = Body mass index, n = number, %g = percentage of girls, yr(s) = year(s), mths = months, wk(s) = week(s), d = day(s), hr(s) = hour(s), min = minute(s), sec = seconds, cnts = counts, kg = kilogram(s), m = meter.

**Supplementary Table 1. Participant and study characteristics, main findings and methodological quality of included studies for body composition (continued).**

| **Reference (Author, year, country)** | **Sample (size (n), age, %girls)** | **Physical activity exposure** | **Outcome measure** | **Main findings*** | **Methodological quality** |
| --- | --- | --- | --- | --- | --- |
| **Longitudinal studies** | | | | | |
| Leppanen et al. (2017), Sweden [13] | n=138, 4.5±0.2yrs, 47%g | PA at baseline:  LPA = 261±28.4min/d,  MPA = 94.6±22.6min/d,  MVPA = 101±24.9min/d,  VPA = 7.2±4.7min/d^2^ | BMI, fat mass index, fat free mass index after 12mths | More MVPA was associated with a higher fat free mass index (0.1kg, 95%CI=0.0, 0.1).  More VPA was associated with a higher fat free mass index (0.4kg, 95%CI=0.2, 0.6) and a higher (0.4kg/m2, 95%CI=0.1, 0.7).  Substituting 5 min/d of light or MPA with 5 min/d of VPA at the age of 4.5yr was associated with higher FFMI and BMI at 5.5 yr. | Moderate |
| Moore et al. (2003), USA [14] | n=103;  low activity level: n=34, 4.2±0.8yrs, 55.9%g;  middle activity level:  n=35, 4.0±0.7yrs, 37.1%g;  high activity level: n=34, 3.9±0.7yrs, 23.5%g | Total PA over 8yrs, per activity level:  Low = 8.5 ± 0.8 cnts/hr,  Middle = 10.2 ± 0.4 cnts/hr,  High = 12.5 ± 1.2 cnts/hr^2^ | BMI and fat mass: triceps, subscapular, suprailiac, abdominal and thigh skinfolds at age 11 | More active children had a lower BMI (18.6±0.6kg/m2) and fat mass (triceps skinfold: 15.1±1.1mm and total skinfolds: 74.1±7.0mm) compared to children with a lower activity level (BMI = 20.3±0.6kg/m^2^; triceps skinfold = 18.6±1.0mm; total skinfold = 95.1±6.8mm). | Moderate |
| DuRant et al. (1994), USA [34] | n=101, 4±0.6yrs, 52%g | PA across one year:  Heartrate = 113.9±7.9 beats/min,  Activity level = 2.0±0.2 (range 1-5)^3^ | Waist/hip ratio at 1yr | PA was associated with waist/hip ratio (r=-21). | Weak |

* = Only significant results, determined at p<0.05, displayed, ^1^ = Reported intervention dose, ^2^ = Valid and reliable measurement/objectively measured physical activity, ^3^ = No valid and/or reliable measurement/subjectively measured physical activity, PA = Physical activity, LPA = Light intensity physical activity, MPA = Moderate intensity physical activity, VPA = Vigorous intensity physical activity, MVPA = Moderate-to-vigorous intensity physical activity, BMI = Body mass index, n = number, %g = percentage of girls, yr(s) = year(s), mths = months, wk(s) = week(s), d = day(s), hr(s) = hour(s), min = minute(s), cnts = counts, kg = kilogram(s), m = meter, mm = millimeter.

**Supplementary Table 2. Participant and study characteristics, main findings and methodological quality of included studies for motor development.**

| **Reference (Author, year, country)** | **Sample (size (n), age, %girls)** | **Physical activity exposure** | **Outcome measure** | **Main findings*** | **Methodological quality** |
| --- | --- | --- | --- | --- | --- |
| **Intervention studies** | | | | | |
| Donath et al. (2015), Switzerland [15] | n=41;  INT: n=22, 4.5±1.0yrs, 55%g;  CON: n=19, 4.4±1.2yrs, 63%g | INT: 2x/wk 30min instructor-led object control training (total 330min) for 6wks + routine activities^1^  CON: 2x/wk instructor-led instructed and supervised training | Object control skills: stationary dribble, catch, kick, overhand throw and underhand roll after 6wks | Children in the intervention group had a higher total score (ŋ_p_^2^=0.1) and dribbling score (ŋ_p_^2^=0.2) compared to the control group. | Weak |
| Goodway and Branta (2003), USA [16] | n=59, 4.7±0.3yrs, 51%g;  INT: n=31, 51.6%g;  CON: n=28, 50%g | INT: 2x/wk 45min researcher-led motor skill program (total 1080min) for 12wks^3^  CON: 7x 45min PA (total 315min) | Gross motor skills: locomotor skills (run, jump, hop, slide, gallop, leap, skip) and object control skills (kick, dribble, throw, catch, strike, bounce) after 12wks | Children in the intervention group had a higher locomotor score (ŋ^2^=0.7) and object control score (ŋ_p_^2^=0.7) compared to control group. | Weak |
| Iivonen et al. (2011), Finland [17] | n=84,  g: 55.4±1.2mths,  b: 55.9±1.1mths, 45.2%g;  INT: n=39, 41%g; CON: n=45, 49%g | INT: 2x/wk 45min teacher-led physical education class (total 2160min) for 8mths^1^  CON: 60min/wk teacher-led physical education class (total 1440min) | Total score gross motor skills: static and dynamic balance (sec), running speed (sec), length of standing broad-jump (cm), manipulative skills (sum of throwing-catching combination, throwing at target and kicking at target; range: 0-25) after 12mths | Boys in the intervention group had a higher running speed (before: 3.3±0.4sec, after: 2.8±0.2sec vs before: 3.3±0.4sec, after: 2.8±0.1sec) compared to the control group. | Weak |
| Ketelhut et al. (2018), Germany [18] | n=172;  INT: n=90, 42.0±4.7mths;  CON: n=82, 41.1±3.8mths | INT: 3x/wk 45min teacher-led structured PA program for 24mths^1^  CON: no physical activity program | Gross motor skills: parallel long jump (cm), running speed (6m sprint; sec), balance (reverse gait, cm) after 24mths | Children in the intervention group had better jumping skills (103.9 vs. 83.7cm), running speed (2.2 vs. 2.6 sec) and balance skills (195 vs. 139 cm) compared to the control group. | Weak |

* = Only significant results, determined at p<0.05, displayed, INT = intervention group, CON = Control group, ^1^ = Reported intervention dose, ^2^ = Valid and reliable measurement/objectively measured physical activity, ^3^ = No valid and/or reliable measurement/subjectively measured physical activity, PA = Physical activity, n = number, %g = percentage of girls, yr(s) = year(s), mths = months, wk(s) = week(s), min = minute(s), sec = seconds, cm = centimeter.

**Supplementary Table 2. Participant and study characteristics, main findings and methodological quality of included studies for motor development (continued).**

| **Reference (Author, year, country)** | **Sample (size (n), age, %girls)** | **Physical activity exposure** | | **Outcome measure** | **Main findings*** | **Methodological quality** |  |
| --- | --- | --- | --- | --- | --- | --- | --- |
| **Intervention studies** | | | | | | | |
| Krombholz et al. (2012), Germany [2] | n=428;  INT: n=211, 55.1±7.3mths, 48%g;  CON: n=217, 54.4±7.8mths, 48.4%g | | INT: 1x/wk 45min teacher-led physical education + minimal 20min/day PA (for example games) for 20mths^1^  CON: 1x/wk 45min teacher-led physical education | Total score motor skills: motor coordination (forward balancing, hopping, backward balancing, lateral jump), fitness (standing broad jump, shuttle run) and manual dexterity (paper-and-pencil test) after 20mths | Children in the intervention group had better motor skills (partial eta^2^=0.06) compared to the control group. | Weak |  |
| Lee & Galloway (2012), USA [19] | n=22;  INT: n=11, 1mths/4.5wks, 45.5%g;  CON: n=11, 1mths/4.5wks, 27.3%g | | INT: 20min/d postural and movement activities by caregiver for 4wks^3^  CON: 20min/d face-to-face communication with caregiver for 4wks (i.e. social interaction) | Total score head control: head in midline in supine, head control: supported sitting, posterior neck muscle, anterior neck muscle, lower from sitting, head in midline without visual simulation [supine], pull to sit, head lift in prone position, lateral head turning to the right and left after 3mths  Behavioral coding: percentage of time in lean, percentage of time upright, percentage of time in turn, percentage of time in pop-up and percentage of time with other postures and movements after 3mths  Kinematic motion analysis: maximum lateral displacement, maximum anterior-posterior displacement, speed of head movements, variability of head 3D speed after 3mths | Children in the intervention group had better overall score (z=-2, r=-0.5), head control (z= -3.4, r=-0.7), more pop-up of the head (z=-3.4, r=-0.7), less turn of the head (z=-3.3, r= -0.7), larger head displacement along y-axis (z=-2.6, r=-0.6) and faster average speeds of head movement (z=-3.7, r=-0.8) compared to the control group. | Weak |  |

* = Only significant results, determined at p<0.05, displayed, INT = intervention group, CON = Control group, ^1^ = Reported intervention dose, ^2^ = Valid and reliable measurement/objectively measured physical activity, ^3^ = No valid and/or reliable measurement/subjectively measured physical activity, PA = Physical activity, MVPA = Moderate-to-vigorous intensity physical activity, n = number, %g = percentage of girls, mths = months, wk(s) = week(s), d = day(s), min = minute(s).

**Supplementary Table 2. Participant and study characteristics, main findings and methodological quality of included studies for motor development (continued).**

| **Reference (Author, year, country)** | **Sample (size (n), age, %girls)** | **Physical activity exposure** | **Outcome measure** | **Main findings*** | **Methodological quality** |  |
| --- | --- | --- | --- | --- | --- | --- |
| **Intervention studies** | | | | | | |
| Lobo & Galloway (2012), USA [20] | n=28;  INT: n=14, 8.9±0.7wks, 50%g;  CON: n=14, 8.7±0.9wks, 50%g | INT: 15min/d handling and positioning experience by caregiver for 18.5d (average 13.8min/d, total 268min)^3^  CON: 15min/d face-to-face interaction with caregiver for 17.5days (average 15min/d, total 267.5min) (i.e. social interaction) | Total score motor skills in four positions: supine, prone, sitting, standing/reaching up to 5mths of age.  Motor milestones: reaching, creeping on hands and knees and walking up to 12mths of age. | Children in the intervention group had greater increases in total motor skills scores (U = 45.5, r = -0.46), prone subscale scores (U = 42.5, r = -0.45), and sitting subscale scores (U = 62, r = -0.34) compared to the control group.  Children in the intervention group were able to transfer objects from one hand to another 2.5wks earlier (U=57.5, r=-0.35), explored the environment by creeping/crawling 5wks earlier (U=58, r= -0.30), walked with support 2.5wks earlier (U=60, r=-0.33) and walked independently for 10ft 6wks earlier (U=57.5, r=-0.35) compared to the control group. | Weak |  |
| Scheffler et al. (2007), Germany [4] | n=127, 3yrs, 55%g | INT: 3x/wk 60min movement program for 24mths^1^  CON: no movement program | Gross motor skills: jumping, running (6m), standing on one leg, balancing forwards, balancing backwards, coordination skills after 24mths | After completion of the intervention, children in the intervention group scored 210% for balancing on the right leg, 187.5% for balancing on the left leg, 137.4% for balancing backwards (all balance), 120.9% jumping, 146.5% on the coordination test, 114.5% running compared to the control group (100%).  *Note: No statistical analysis conducted.* | Weak |  |

* = Only significant results, determined at p<0.05, displayed, INT = intervention group, CON = Control group, ^1^ = Reported intervention dose, ^2^ = Valid and reliable measurement/objectively measured physical activity, ^3^ = No valid and/or reliable measurement/subjectively measured physical activity, PA = Physical activity, n = number, %g = percentage of girls, yr(s) = year(s), mths = months, wk(s) = week(s), d = day(s), min = minute(s).

**Supplementary Table 2. Participant and study characteristics, main findings and methodological quality of included studies for motor development (continued).**

| **Reference (Author, year, country)** | **Sample (size (n), age, %girls)** | **Physical activity exposure** | **Outcome measure** | **Main findings*** | **Methodological quality** |
| --- | --- | --- | --- | --- | --- |
| **Intervention studies** | | | | | |
| Sigmundsson and Hopkins (2009), Norway [21] | n=38;  INT: n=19, 4.7±0.2yrs;  CON: n=19, 4.5±0.2yrs | INT: 2 hr/wk baby swimming during the 1^st^ year of life^1^  CON: No swimming | Total score motor skills: manual dexterity (posting coins, threading bead, bicycle trail), object control skills (catching a bean bag, rolling ball into goal), balance (one-leg balance, jumping over cord, walking heels raised) at age 4yrs | Children in the intervention group had better one-leg balance at age 4yrs (0.02±0.1 vs 0.4±0.8; 95%CI=-0.8, 0.01) compared to the control group. | Weak |
| Venetsanou & Kambas (2004), Greece [22] | n=66, 59.8±6.4mths, 45.5%g;  INT: n=28;  CON: n=38 | INT: 2x/wk 45min teacher-led dance program (traditional Greek dance) for 20wks^1^  CON: Regular Kindergarten curriculum | Total score motor skills: jumping with both feet, forwards and backwards toe-to-heel walking, making dots with pencil (tapping), picking up handkerchief using toes, jumping sideways, catching a stick, carrying three balls into box, overhead toss, picking up 40 matches and putting them in a box, stepping through hoop, one-legged jump, catching rubber-ring, stride jumping with rebound and arms clapping above head, standing jump over rope, body rolling along vertical axis, raise-sitting with a ball from squat position, jumping while making 90 degrees turns after 20wks. | Children in the intervention group had better gross motor skills (mean difference 3.7, range 0-34) compared to the control group. | Weak |

* = Only significant results, determined at p<0.05, displayed, INT = intervention group, CON = Control group, ^1^ = Reported intervention dose, ^2^ = Valid and reliable measurement/objectively measured physical activity, ^3^ = No valid and/or reliable measurement/subjectively measured physical activity, PA = Physical activity, n = number, %g = percentage of girls, yr(s) = year(s), mths = months, wk(s) = week(s), hr(s) = hour(s), min = minute(s).

**Supplementary Table 2. Participant and study characteristics, main findings and methodological quality of included studies for motor development (continued).**

| **Reference (Author, year, country)** | **Sample (size (n), age, %girls)** | **Physical activity exposure** | **Outcome measure** | **Main findings*** | **Methodological quality** |  |
| --- | --- | --- | --- | --- | --- | --- |
| **Longitudinal studies** | | | | | | |
| Kuo et al. (2008), Taiwan [23] | n=216, newborn, 47%g | Wakeful prone experience (yes/no) and duration (categories a: 0 min/d, b: 1-19 min/d, c: 20-30 min/d, ≥40 min/d) at age 4mths^3^  Wakeful prone preference (yes/no) at age 6mths | Total score gross motor skills and total score fine motor skills at age 6mths.  Motor milestones: rolling, crawling-on-abdomen, crawling-on-hands and feet, transferring objects, sitting, walking up to 24mths. | Infants with prone experience at age 4mths crawled on abdomen earlier (7.0 vs. 7.3mths) compared to infants without prone experience.  Higher duration of prone position at age 4mths was associated with achieving milestones earlier: rolling (a: 5mths, b: 4mths, c: 5mths, d: 3.5mths), crawling-on-abdomen (a: 7.3mths, b: 7mths, c: 6mths, d: 5mths), crawling-on-4s (a: 9mths, b: 8mths, c: 7.3mths, d: 6.5mths), and sitting (a: 7mths, b: 7mths, c: 6mths, d: 6mths) compared to lower duration of prone position. | Moderate |  |

* = Only significant results, determined at p<0.05, displayed, ^1^ = Reported intervention dose, ^2^ = Valid and reliable measurement/objectively measured physical activity, ^3^ = No valid and/or reliable measurement/subjectively measured physical activity, n = number, %g = percentage of girls, mths = months, d = day(s), min = minute(s).

**Supplementary Table 3. Participant and study characteristics, main findings and methodological quality of included studies for cognitive development.**

| **Reference (Author, year, country)** | **Sample (size (n), age, %girls)** | **Physical activity exposure** | **Outcome measure** | **Main findings*** | **Methodological quality** |
| --- | --- | --- | --- | --- | --- |
| **Intervention studies** | | | | | |
| Holmes et al. (2006), USA [24] | n=27, 55.6±4mths, 66.7%g | INT1= 10min recess;  INT2= 20min recess;  INT3= 30min recess^1^ | Attention and concentration  (0= inattentive, 1=attentive) directly after recess | Children had better attention/concentration after 20mins of recess (0.8±0.2) followed by 10mins of recess (0.8±0.2) and 30mins of recess (0.3±0.4). | Weak |
| Kirk et al. (2014), USA [25] | n=72, 3.9±0.1yrs, 53%g;  INT: n=51, 3.8±0.1yrs, 56.9%g;  CON: n=21, 3.9±0.1yrs, 42.9%g; | INT: 2x/day 15min teacher-led MVPA during learning for 6mths (145±10min/wk)^3^  CON: regular classroom instruction | Early literacy and language: picture naming, alliteration and rhyming (measures as correct words per 2min) after 6mths | Children in the intervention group had better scores for alliteration (INT: 1.0±0.3 words; CON: 0.5±0.3 words) and picture naming (INT: 24.8±3.4 words; CON: 19.3±2.3 words) compared to the control group. | Weak |
| Kirk and Kirk (2016), USA [26] | n=54, 4.1±0.2yrs, 69%g | INT: 2x/d 30min teacher-led PA during learning for 8mths (activity level at 4mths = 3.5±0.4 and at 8mths= 3.5±0.4; range 1-5)^3^  CON: regular classroom instruction (activity level at 4mths = 2.5±0.4 and at 8mths = 2.6±0.5; range 1-5) | Early literacy and language: picture naming, alliteration and rhyming (measures as correct words per 2min) after 8mths | Children in the intervention group had better scores for alliteration (INT: 52±16 words; CON: 13±5 words) and rhyming (INT: 173±12 words; CON: 28±8 words) (INT: 24.8±3.4 words; CON: 19.3±2.3 words) compared to the control group. | Weak |

* = Only significant results, determined at p<0.05, displayed, INT = intervention group, CON = Control group, ^1^ = Reported intervention dose, ^2^ = Valid and reliable measurement/objectively measured physical activity, ^3^ = No valid and/or reliable measurement/subjectively measured physical activity, PA = Physical activity, MVPA = Moderate-to-vigorous intensity physical activity, n = number, %g = percentage of girls, yr(s) = year(s), mths = months, wk(s) = week(s), d = day(s), hr(s) = hour(s), min = minute(s).

**Supplementary Table 3. Participant and study characteristics, main findings and methodological quality of included studies for cognitive development (continued).**

| **Reference (Author, year, country)** | **Sample (size (n), age, %girls)** | **Physical activity exposure** | **Outcome measure** | **Main findings*** | **Methodological quality** |  |
| --- | --- | --- | --- | --- | --- | --- |
| **Intervention studies** | | | | | | |
| Mavilidi et al. (2015), Australia [27] | n=111, 4.9±0.6yrs, 49%g  INT1: n=31,  INT2: n=23,  CON: n=57 | INT1: 2x/wk 15min researcher-led integrated PA during learning for 4wks (938.1±552.8cnts/min)^2^;  INT2: 2x/wk 15min researcher-led non-integrated PA during learning for 4wks (807.6±552.8cnts/min);  CON: 2x/wk 15min researcher-led sitting during learning for 4wks (534.7±311.3cnts/min) | Language: Italian words free-call and cued recall test | Children in the integrated PA intervention (M=1.7 words, SD=1.3) recalled more Italian words during free-call compared to the non-integrated PA intervention (M=1.0 words, SD=1) and the control group (M=0.8 words, SD=0.9).  Children in the integrated PA intervention (M=4 words, SD=2.3) recalled more Italian words during cued recall compared to the control group (M=2.1 words, SD=1.5).  Children in the non-integrated PA intervention (M=3.2 words, SD=1.6) recalled more Italian words during cued recall compared to the control group (M=2.1 words, SD=1.5). | Weak |  |
| Mavilidi et al. (2016), Australia [28] | n=87, 4.9±0.6yrs, 50%g;  INT1: n=28,  INT2: n=29,  CON: n=30 | INT1: 3x 10min researcher-led integrated PA during learning (1038.5±489cnts/min) over 8d^2^;  INT2: 3x 10min researcher-led non-integrated PA during learning (1746.9±622.2cnts/min) over 8days;  CON: 3x 10min researcher-led sitting during learning (603.7±487.8cnts/min) over 8days | Geography: naming continents and matching animals after 5wks | Children in the integrated PA intervention (M=16.1 names/matches, SD=3.4) had better geography scores compared to the non-integrated PA intervention (M=14.5 names/matches, SD=3.8) and the control group (M=11.4 names/matches, SD=3.6).  Children in the non-integrated PA intervention (M=14.5 names/matches, SD=3.8) had better geography scores compared to the control group (M=11.4 names/matches, SD=3.6). | Weak |  |

* = Only significant results, determined at p<0.05, displayed, INT = intervention group, CON = Control group, ^1^ = Reported intervention dose, ^2^ = Valid and reliable measurement/objectively measured physical activity, ^3^ = No valid and/or reliable measurement/subjectively measured physical activity, PA = Physical activity, MVPA = Moderate-to-vigorous intensity physical activity, n = number, %g = percentage of girls, yr(s) = year(s), mths = months, wk(s) = week(s), d = day(s), hr(s) = hour(s), min = minute(s), sec = seconds, cnts = counts.

**Supplementary Table 3. Participant and study characteristics, main findings and methodological quality of included studies for cognitive development (continued).**

| **Reference (Author, year, country)** | **Sample (size (n), age, %girls)** | **Physical activity exposure** | **Outcome measure** | **Main findings*** | **Methodological quality** |  |
| --- | --- | --- | --- | --- | --- | --- |
| **Intervention studies** | | | | | | |
| Mavilidi et al. (2017), Australia [29] | n=86, 4.9±0.5yrs, 50%g | INT1: 1x/wk 10min researcher-led integrated PA during learning for 4wks (878.2cnts/min)^2^;  INT2: 1x/wk 10min researcher-led non-integrated PA during learning for 4wks (1117cnts/min);  CON: 1x/wk 10min researcher-led sitting during learning for 4wks (530.3cnts/min); | Science: planets after 10wks | Children in the integrated PA intervention (M=14.1 planets, SE=0.7) had better geography scores compared to the non-integrated PA intervention (M=10.1 planets, SE=0.8) and the control group (M=5.3 planets, SE=0.8). | Weak |  |
| Mavilidi et al. (2018), Australia [30] | n=115, 4.7±0.5yrs, 47.5%g | INT1: 1x/wk 15min integrated PA during learning for 4wks (1228.3cnts/min)^2^;  INT2: 1x/wk 15min non-integrated PA during learning for 4wks (1008.6cnts/min);  CON: 1x/wk 15min sitting during learning for 4wks (462.2cnts/min) | Numeracy: counting, number line estimation, block counting, numerical magnitude comparison, numerical identification after 10wks | Children in the integrated PA intervention (M=35.3 correct, SE=2.0) had higher numeracy scores compared to the non-integrated PA intervention (M=27.2 correct, SE=2.1) and the control group (M=22.1 correct, SE=2.1). | Weak |  |
| Palmer et al. (USA), 2013 [31] | n=16, 49.4±5.3mths, 18.8%g | INT: 1x 30min researcher-led movement program (17.6±3.5min MVPA)^2^  CON: 1x 30min sedentary activity (2.4±2.0min MVPA) | Sustained attention (number of commissions) and response inhibition (number of omissions; range 0-60) directly after the movement program | Children in the intervention group had greater sustained attention (25.6±12.3) compared to the control group (44.3±28.7). | Weak |  |

* = Only significant results, determined at p<0.05, displayed, INT = intervention group, CON = Control group, ^1^ = Reported intervention dose, ^2^ = Valid and reliable measurement/objectively measured physical activity, ^3^ = No valid and/or reliable measurement/subjectively measured physical activity, PA = Physical activity, MVPA = Moderate-to-vigorous intensity physical activity, n = number, %g = percentage of girls, yr(s) = year(s), mths = months, wk(s) = week(s), hr(s) = hour(s), min = minute(s), sec = seconds, cnts = counts.

**Supplementary Table 3. Participant and study characteristics, main findings and methodological quality of included studies for cognitive development (continued).**

| **Reference (Author, year, country)** | **Sample (size (n), age, %girls)** | **Physical activity exposure** | **Outcome measure** | **Main findings*** | **Methodological quality** |
| --- | --- | --- | --- | --- | --- |
| **Intervention studies** | | | | | |
| Webster et al. (2015), USA [32] | n=118, 3.8±0.7yrs, 53.4%g | INT: 2x/d 10min teacher-led MVPA classroom break (3.2min MVPA, 29.7%)^2^  CON: 2x/d 10min typical classroom instruction (0.1min MVPA, 0.7&) | Concentration: percentage of time spent on task directly after activity break | No differences between groups at follow-up. | Weak |
| **Longitudinal studies** | | | | | |
| Gialamas et al. (2019), Australia | n=4253, 8.8±2.6mths, 48.8%g | Time investment in PA:  Wave 1, 0-1yr old= 2.0±2.0hr/d,  Wave 2, 2-3yr old= 1.9±1.4hr/d^3^ | Receptive vocabulary at ages 4-5yrs | Every additional 1hr/day of time investment in PA at ages 0-1yrs leads to a decrease in receptive vocabulary score at ages 4-5yrs (-0.02%; 95%CI= -0.19-0.14).  Every additional 1hr/day of time investment in PA at ages 2-3yrs leads to a decrease in receptive vocabulary score at ages 4-5yrs (-0.10%; 95%CI= -0.33-0.13). | Moderate |

* = Only significant results, determined at p<0.05, displayed, INT = intervention group, CON = Control group, ^1^ = Reported intervention dose, ^2^ = Valid and reliable measurement/objectively measured physical activity, ^3^ = No valid and/or reliable measurement/subjectively measured physical activity, PA = Physical activity, MVPA = Moderate-to-vigorous intensity physical activity, n = number, %g = percentage of girls, yr(s) = year(s), mths = months, d = day(s), hr(s) = hour(s), min = minute(s).

**Supplementary Table 4. Participant and study characteristics, main findings and methodological quality of included studies for cardiovascular outcomes.**

| **Reference (Author, year, country)** | **Sample (size (n), age, %girls)** | **Physical activity exposure** | **Outcome measure** | **Main findings*** | **Methodological quality** |
| --- | --- | --- | --- | --- | --- |
| **Intervention studies** | | | | | |
| Ketelhut et al. (2018), Germany [18] | n=172;  INT: n=90, 42±4.7mths; CON: n=82, 41.1±3.8mths | INT: 3x/wk 45min teacher-led structured PA program for 24mths^1^  CON: no physical activity program | Blood pressure in rest and during exercise | Children in the intervention group had a lower diastolic blood pressure in rest (65.7 vs. 68.1mmHg) and during exercise (62 vs 68.8mmHg) compared to the control group. | Weak |
| Scheffler et al. (2007), Germany [4] | n=127, 3yrs, 55%g | INT: 3x/wk 60min teacher-led movement program for 24mths^1^  CON: No movement program | Blood pressure during exercise, 1min after exercise and 3min after exercise | After completion of the intervention, children in the intervention group had a blood pressure of 62±11.2 mmHg during exercise, 65.1 ±9.4mmHg 1min after exercise and 64.4±9.2mmHg 3mins after exercise and children in the control group had a blood pressure of 68.8±11.1mmHg during exercise, 68.1±9.2mmHg 1min after exercise and 67.6±8.3mmHg 3mins after exercise.  *Note: No statistical analysis conducted.* | Weak |
| **Longitudinal studies** | | | | | |
| Metcalf et al. (2008), UK | N=212, 4.9yr, 46.7%g  g: 4.9yrs (range 4.9 to 5),  b: 4.92yrs (range 4.9 to 5) | PA (≥3 METs) over 48mths:  g = 45min/d  b = 57min/d^2^ | Metabolic markers separate and as Z-score: insulin resistance, triglycerides, cholesterol/HDL ratio and mean arterial blood pressure | Higher levels of PA were associated with lower metabolic z-score (r=-0.2) and triglycerides (r=-0.3) in girls, and lower mean blood pressure (r=-0.2) in boys. | High |

* = Only significant results, determined at p<0.05, displayed, INT = intervention group, CON = Control group, ^1^ = Reported intervention dose, ^2^ = Valid and reliable measurement/objectively measured physical activity, ^3^ = No valid and/or reliable measurement/subjectively measured physical activity, PA = Physical activity, MET = Metabolic equivalent of task, n = number, %g = percentage of girls, yr(s) = year(s), mths = months, wk(s) = week(s), d = day(s), min = minute(s), mmHg = millimeters of mercury.

**Supplementary Table 4. Participant and study characteristics, main findings and methodological quality of included studies for cardiovascular outcomes (continued).**

| **Reference (Author, year, country)** | **Sample (size (n), age, %girls)** | **Physical activity exposure** | **Outcome measure** | **Main findings*** | **Methodological quality** |
| --- | --- | --- | --- | --- | --- |
| **Longitudinal studies** | | | | | |
| Metcalf et al. (2009), United Kingdom [33] | n=213, 4.9±0.3yrs, 45.1%g | PA across 3yrs:  Total PA:  g = 34.9*10^5^cnts/d,  b = 38.1*10^5^cnts/d;  Average MVPA:  g = 45.1min/d,  b = 56min/d^2^ | Metabolic markers: serum insulin, glucose, adiponectin, leptin, hsCRP at 8yrs of age | A higher level of PA was associated with lower levels of adiponectin (r=-0.3).  A higher level of MVPA was associated with lower levels of adiponectin (r=-0.3). | High |
| Proudfoot et al. (2019), Canada | n=418, 4.5±0.9yrs, 49.8%g | Total PA assessed over 3yrs:  Y1 = 256.5±37.8min/d  Y2 = 255.9±37.2min/d  Y3 = 256.5±40.7min/d^2^  MVPA assessed over 3yrs:  Y1 = 96.4±21.7min/d  Y2 = 99.5±21.7min/d  Y3 = 102.3±24.4min/d | Cardiovascular health indicators: cardiovascular fitness (exercise time on a maximal treadmill test and 1-minute HRR), resting arterial stiffness (whole-body PWV and carotid b stiffness index), and seated systolic blood pressure: SBP over 3yrs. | Total PA had positive main effects on treadmill time (b=0.004, SE=0.001), HRR (b=0.05, SE=0.01) and PWV (b=-0.001, SE=0.0004). MVPA had positive main effects on treadmill time (b=0.008, SE=0.002) and HRR (b=0.08, SE=0.02). MVPA associated with slower increase in PWV (b=-0.002, SE=0.0007) and b stiffness index (b=-0.003, SE=0.001). | High |

* = Only significant results, determined at p<0.05, displayed, ^1^ = Reported intervention dose, ^2^ = Valid and reliable measurement/objectively measured physical activity, ^3^ = No valid and/or reliable measurement/subjectively measured physical activity, PA = Physical activity, MVPA = Moderate-to-vigorous intensity physical activity, n = number, %g = percentage of girls, g = girls, b = boys, yr(s) = year(s), d = day(s), min = minute(s), cnts = counts, hsCRP = high-sensitivity C-reactive protein, HRR = Heart rate recovery, PWV = Pulse wave velocity.

**Supplementary Table 4. Participant and study characteristics, main findings and methodological quality of included studies for cardiovascular outcomes (continued).**

| **Reference (Author, year, country)** | **Sample (size (n), age, %girls)** | **Physical activity exposure** | **Outcome measure** | **Main findings*** | **Methodological quality** |
| --- | --- | --- | --- | --- | --- |
| **Longitudinal studies** | | | | | |
| Leppanen et al. (2017), Sweden [13] | n=138, 4.5±0.2yrs, 47%g | PA at baseline:  LPA = 261±28.4min/d,  MPA = 94.6±22.6min/d,  VPA = 7.16±4.69min/d,  MVPA = 101±24.9min/d^2^ | Physical fitness: 20m shuttle run test (laps), handgrip test (kg), standing long jump (cm), 4x10m shuttle run (sec) at 12mths | MVPA was associated with improved physical fitness: 20m shuttle run test (0.36 lap; 95%CI=0.08,0.64), standing long jump (1.37cm; 95%CI=0.19,2.54), 4x10m shuttle run (-0.11sec; 95%CI=-0.21,-0.01).  VPA was associated with improved physical fitness: 20-m shuttle run test (1.05 lap; 95%CI=0.20,1.89), standing long jump (5.86cm; 95%CI=2.45,9.26), 4x10m shuttle run (-0.32sec; 95%CI=-0.61,-0.03).  Substituting 5 min/d of light or MPA with 5 min/d of VPA at the age of 4.5yrs was associated with better handgrip strength and standing long jump. | Moderate |
| DuRant et al. (1994), USA [34] | n=101, 4±0.6yrs, 52%g | PA across one year:  Heartrate = 113.9±7.9 beats/min,  Activity level = 2.0±0.2 (range 1-5)^3^ | Serum lipids and lipoproteins: triglycerides, total serum cholesterol, high-density and low-density lipoprotein after 1yr | PA was not associated with serum lipids or lipoproteins. | Weak |

* = Only significant results, determined at p<0.05, displayed, ^1^ = Reported intervention dose, ^2^ = Valid and reliable measurement/objectively measured physical activity, ^3^ = No valid and/or reliable measurement/subjectively measured physical activity, PA = Physical activity, LPA = Light intensity physical activity, MPA = Moderate intensity physical activity, VPA = Vigorous intensity physical activity, MVPA = Moderate-to-vigorous intensity physical activity, n = number, %g = percentage of girls, yr(s) = year(s), mths = months, d = day(s), min = minute(s), sec = seconds, cnts = counts, cm = centimeter, kg = kilogram.

**Supplementary Table 5. Participant and study characteristics, main findings and methodological quality of included studies for social-emotional development.**

| **Reference (Author, year, country)** | **Sample (size (n), age, %girls)** | **Physical activity exposure** | **Outcome measure** | **Main findings*** | **Methodological quality** |
| --- | --- | --- | --- | --- | --- |
| **Longitudinal studies** | | | | | |
| Gialamas et al. (2019), Australia | n=4253, 8.8±2.6mths, 48.8%g | Time investment in PA over 5yrs:  Wave 1, 0-1yr old= 2.0±2.0hr/d,  Wave 2, 2-3yr old= 1.9±1.4hr/d,  Wave 3, 4-5yr old=1.6±1.3hr/d^3^ | Externalizing problem behaviors (hyperactivity and conduct problems) reported by parents and teachers at ages 4-5yrs | Every additional 1hr/d of time investment in PA at ages 0-1yrs leads to an increase in externalizing behavior reported by parents (0.2%; 95%CI= 1.0-1.3) and decrease reported by teachers at (1.1%; 95%CI= -2.9-0.7) ages 4-5yrs  Every additional 1hr/d of time investment in PA at ages 2-3yrs leads to an increase in externalizing behavior reported by parents (1.8%; 95%CI= 0.2-3.3) and increase reported by teachers (2.1%; 95%CI= -0.2-4.4) at ages 4-5yrs. | Moderate |
| Vella et al. (2015), Australia [35] | n=2785, 4-5yrs, 47.8%g | PA at ages 4-5yrs = 76.7±73.7hr/wk^3^ | Health related quality of life at ages 12-13yrs | PA was not associated with health related quality of life. | Moderate |

* = Only significant results, determined at p<0.05, displayed, ^1^ = Reported intervention dose, ^2^ = Valid and reliable measurement/objectively measured physical activity, ^3^ = No valid and/or reliable measurement/subjectively measured physical activity, PA = Physical activity, n = number, %g = percentage of girls, yr(s) = year(s), mths = months, wk(s) = week(s), d = day(s), hr(s) = hour(s), min = minute(s).

**Supplementary Table 6. Participant and study characteristics, main findings and methodological quality of included studies for bone health.**

| **Reference (Author, year, country)** | **Sample (size (n), age, %girls)** | **Physical activity exposure** | **Outcome measure** | **Main findings*** | **Methodological quality** |
| --- | --- | --- | --- | --- | --- |
| **Longitudinal studies** | | | | | |
| Wosje et al. (2009), USA [36] | n=215, 3.3-3.8yrs, 49%g | Outdoor play ages 3-7yr by ethnicity:  Afro-American:  g = 1.5±0.1hr/d,  b = 1.5±0.2hr/d;  Caucasian:  g = 1.6±0.1hr/d,  b = 1.8±0.06hr/d^2^  PA ages 3-7yr by ethnicity:  Afro-American:  g = 471±86cnts/min,  b = 513±83cnts/min;  Caucasian:  g = 541±89cnts/min,  b = 598±110 cnts/min | Bone density at age 7yrs | PA was not associated with bone density. | High |
| Clark et al. (2008), UK [37] | n=2692, 4.5yrs, 52.6%g | Outdoor play in summer at baseline in hr/wk^3^ | Fractures at age 9.8yrs | Spending more than 28 hr/wk in outside play is associated with an increased chance of fractures (OR = 2.4; 95%CI=1.6, 3.6). | Moderate |

* = Only significant results, determined at p<0.05, displayed, ^1^ = Reported intervention dose, ^2^ = Valid and reliable measurement/objectively measured physical activity, ^3^ = No valid and/or reliable measurement/subjectively measured physical activity, PA = Physical activity, n = number, %g = percentage of girls, g = girls, b = bpys, yr(s) = year(s), wk(s) = week(s), d = day(s), hr(s) = hour(s), min = minute(s), cnts = counts.

**References**

1. Goldfield, G.S., et al., *Effects of Child Care Intervention on Physical Activity and Body Composition.* Am J Prev Med, 2016. **51**(2): p. 225-231.

2. Krombholz, H., *The impact of a 20-month physical activity intervention in child care centers on motor performance and weight in overweight and healthy-weight preschool children.* Perceptual & Motor Skills, 2012. **115**(3): p. 919-932.

3. Mo-suwan, L., et al., *Effects of a controlled trial of a school-based exercise program on the obesity indexes of preschool children.* Am J Clin Nutr, 1998. **68**(5): p. 1006-11.

4. Scheffler, C., K. Ketelhut, and I. Mohasseb, *Does physical education modify the body composition?--results of a longitudinal study of pre-school children.* Anthropol Anz, 2007. **65**(2): p. 193-201.

5. Carter, P.J., et al., *Longitudinal analysis of sleep in relation to BMI and body fat in children: the FLAME study.* Bmj, 2011. **342**: p. d2712.

6. De Coen, V., et al., *Risk factors for childhood overweight: a 30-month longitudinal study of 3- to 6-year-old children.* Public Health Nutr, 2014. **17**(9): p. 1993-2000.

7. Jago, R., et al., *BMI from 3-6 y of age is predicted by TV viewing and physical activity, not diet.* International Journal of Obesity, 2005. **29**(6): p. 557-564.

8. Metcalf, B.S., et al., *Physical activity at the government-recommended level and obesity-related health outcomes: a longitudinal study (Early Bird 37).* Arch Dis Child, 2008. **93**(9): p. 772-7.

9. Wells, J.C. and P. Ritz, *Physical activity at 9-12 months and fatness at 2 years of age.* Am J Hum Biol, 2001. **13**(3): p. 384-9.

10. Saldanha-Gomes, C., et al., *Prospective associations between energy balance-related behaviors at 2 years of age and subsequent adiposity: the EDEN mother-child cohort.* Int J Obes (Lond), 2017. **41**(1): p. 38-45.

11. Butte, N.F., et al., *Role of physical activity and sleep duration in growth and body composition of preschool-aged children.* Obesity (Silver Spring), 2016. **24**(6): p. 1328-35.

12. Ip, E.H., et al., *Physical Activity States of Preschool-Aged Latino Children in Farmworker Families: Predictive Factors and Relationship With BMI Percentile.* Journal of Physical Activity & Health, 2016. **13**(7): p. 726-732.

13. Leppänen, M.H., et al., *Longitudinal Physical Activity, Body Composition, and Physical Fitness in Preschoolers.* Medicine & Science in Sports & Exercise, 2017. **49**(10): p. 2078-2085.

14. Moore, L.L., et al., *Does early physical activity predict body fat change throughout childhood?* Preventive Medicine: An International Journal Devoted to Practice and Theory, 2003. **37**(1): p. 10-17.

15. Donath, L., et al., *Fundamental movement skills in preschoolers: A randomized controlled trial targeting object control proficiency.* Child: Care, Health and Development, 2015. **41**(6): p. 1179-1187.

16. Goodway, J.D. and C.F. Branta, *Influence of a motor skill intervention on fundamental motor skill development of disadvantaged preschool children.* Res Q Exerc Sport, 2003. **74**(1): p. 36-46.

17. Iivonen, S., A. Sääkslahti, and K. Nissinen, *The development of fundamental motor skills of four‐ to five‐year‐old preschool children and the effects of a preschool physical education curriculum.* Early Child Development and Care, 2011. **181**(3): p. 335-343.

18. Ketelhut, K., I. Mohasseb, and R.G. Ketelhut, *Two years of regular exercise decreases blood pressure and improves motor skills in early childhood.* Sport Sciences for Health, 2018. **14**(3): p. 571-578.

19. Lee, H.-M. and J.C. Galloway, *Early Intensive Postural and Movement Training Advances Head Control in Very Young Infants.* Physical Therapy, 2012. **92**(7): p. 935-947.

20. Lobo, M.A. and J.C. Galloway, *Enhanced handling and positioning in early infancy advances development throughout the first year.* Child Development, 2012. **83**(4): p. 1290-1302.

21. Sigmundsson, H. and B. Hopkins, *Baby swimming: exploring the effects of early intervention on subsequent motor abilities.* Child Care Health Dev, 2010. **36**(3): p. 428-30.

22. Venetsanou, F. and A. Kambas, *How can a traditional Greek dances programme affect the motor proficiency of pre‐school children?* Research in Dance Education, 2004. **5**(2): p. 127-138.

23. Kuo, Y.-L., et al., *The influence of wakeful prone positioning on motor development during the early life.* Journal of Developmental and Behavioral Pediatrics, 2008. **29**(5): p. 367-376.

24. Holmes, R.M., A.D. Pellegrini, and S.L. Schmidt, *The effects of different recess timing regimens on preschoolers' classroom attention.* Early Child Development and Care, 2006. **176**(7): p. 735-743.

25. Kirk, S.M., et al., *Using Physical Activity to Teach Academic Content: A Study of the Effects on Literacy in Head Start Preschoolers.* Early Childhood Education Journal, 2014. **42**(3): p. 181-189.

26. Kirk, S.M. and E.P. Kirk, *Sixty Minutes of Physical Activity per Day Included Within Preschool Academic Lessons Improves Early Literacy.* J Sch Health, 2016. **86**(3): p. 155-63.

27. Mavilidi, M.-F., et al., *Effects of Integrated Physical Exercises and Gestures on Preschool Children’s Foreign Language Vocabulary Learning.* Educational Psychology Review, 2015. **27**(3): p. 413-426.

28. Mavilidi, M.-F., et al., *Infusing Physical Activities Into the Classroom: Effects on Preschool Children's Geography Learning.* Mind, Brain, and Education, 2016. **10**(4): p. 256-263.

29. Mavilidi, M.-F., et al., *Effects of Integrating Physical Activities Into a Science Lesson on Preschool Children's Learning and Enjoyment.* Applied Cognitive Psychology, 2017. **31**(3): p. 281-290.

30. Mavilidi, M.-F., et al., *Immediate and delayed effects of integrating physical activity into preschool children’s learning of numeracy skills.* Journal of Experimental Child Psychology, 2018. **166**: p. 502-519.

31. Palmer, K.K., M.W. Miller, and L.E. Robinson, *Acute exercise enhances preschoolers' ability to sustain attention.* J Sport Exerc Psychol, 2013. **35**(4): p. 433-7.

32. Webster, E.K., D.D. Wadsworth, and L.E. Robinson, *Preschoolers' time on-task and physical activity during a classroom activity break.* Pediatr Exerc Sci, 2015. **27**(1): p. 160-7.

33. Metcalf, B.S., et al., *Objectively measured physical activity and its association with adiponectin and other novel metabolic markers: a longitudinal study in children (EarlyBird 38).* Diabetes Care, 2009. **32**(3): p. 468-73.

34. DuRant, R.H., et al., *Association among serum lipid and lipoprotein concentrations and physical activity, physical fitness, and body composition in young children.* J Pediatr, 1993. **123**(2): p. 185-92.

35. Vella, S.A., C.A. Magee, and D.P. Cliff, *Trajectories and predictors of health-related quality of life during childhood.* The Journal of Pediatrics, 2015. **167**(2): p. 422-427.

36. Wosje, K.S., et al., *Adiposity and TV viewing are related to less bone accrual in young children.* J Pediatr, 2009. **154**(1): p. 79-85.e2.

37. Clark, E.M., A.R. Ness, and J.H. Tobias, *Vigorous Physical Activity Increases Fracture Risk in Children Irrespective of Bone Mass: A Prospective Study of the Independent Risk Factors for Fractures in Healthy Children.* Journal of Bone & Mineral Research, 2008. **23**(7): p. 1012-22.
