# Supplementary material for: Physical activity and prospective associations with indicators of health and development in children aged <5 years: a systematic review
Source: Int J Behav Nutr Phys Act. 2021 Jan 7;18:6. doi: 10.1186/s12966-020-01072-w (PMC7791660; doi:10.1186/s12966-020-01072-w)
Supplement: Supplementary file 4 — Additional file 4: Table S7. This additional file includes Table S7 that displays the methodological quality of all included studies. [file 12966_2020_1072_MOESM4_ESM.docx]

**Supplementary Table 7. Methodological quality of included studies.**

| **Study** | **A Selection Bias** | **B Study Design** | **C Confounders** | **D Blinding^1^** | **E Data Collection Methods** | **F Withdrawals and Drop-outs** | **G Intervention Integrity^1^** | **H Analysis** | **Overall Score** | |
| --- | --- | --- | --- | --- | --- | --- | --- | --- | --- | --- |
| **Body Composition** | | | | | | | | | | |
| Carter et al. 2011 | P | G | G | NA | G | G | NA | G | | High |
| De Coen et al. 2013 | P | G | F | NA | F | F | NA | G | | High |
| Jago et al. 2005 | P | G | F | NA | G | F | NA | G | | High |
| Metcalf et al. 2008 | F | G | G | NA | G | F | NA | F | | High |
| Saldanha-Gomes et al. 2017 | F | G | P | NA | F | F | NA | G | | High |
| Wells & Ritz 2001 | P | G | F | NA | G | F | NA | G | | High |
| Butte et al. 2016 | P | G | P | NA | G | F | NA | F | | Moderate |
| Ip et al. 2016 | P | G | G | NA | G | F | NA | P | | Moderate |
| Leppanen et al. 2017 | P | G | P | NA | G | G | NA | G | | Moderate |
| Moore et al. 2003 | P | G | G | NA | G | F | NA | P | | Moderate |
| DuRant et al. 1993 | P | G | P | N/A | G | F | N/A | P | | Weak |
| Goldfield et al. 2016 | P | G | F | P | G | G | P | G | | Weak |
| Krombholz et al. 2012 | P | F | F | P | F | F | P | P | | Weak |
| Mo-suwam et al. 1998 | P | G | F | P | F | G | P | F | | Weak |
| Scheffler et al. 2007 | P | F | P | P | F | P | P | P | | Weak |
| **Motor Development** | | | | | | | | | | |
| Kuo et al. 2008 | P | G | F | NA | F | F | NA | P | | Moderate |
| Donath et al. 2015 | P | G | G | P | F | F | P | G | | Weak |
| Goodway & Branta 2003 | P | F | F | P | F | P | P | F | | Weak |
| Iivonen et al. 2011 | P | F | F | P | P | P | p | G | | Weak |
| Ketelhut et al. 2018 | P | G | G | P | P | F | P | P | | Weak |
| Krombholz 2012 | P | F | F | P | P | F | P | F | | Weak |
| Lee & Galloway 2012 | P | G | F | G | P | G | P | G | | Weak |
| Lobo & Galloway 2012 | P | G | P | G | G | G | P | G | | Weak |
| Scheffler et al. 2007 | P | F | P | P | P | P | P | P | | Weak |
| Sigmundsson and Hopkins 2009 | P | F | P | P | F | P | P | F | | Weak |
| Venetsanou & Kambas 2004 | P | F | F | P | P | P | P | G | | Weak |

^1^ Items D and G were not applicable for longitudinal studies, NA = Not applicable.

**Supplementary Table 7. Methodological quality of included studies (continued).**

| **Study** | **A Selection Bias** | **B Study Design** | **C Confounders** | **D Blinding^1^** | **E Data Collection Methods** | **F Withdrawals and Drop-outs** | **G Intervention Integrity^1^** | **H Analysis** | **Overall Score** | |
| --- | --- | --- | --- | --- | --- | --- | --- | --- | --- | --- |
| **Cognitive Development** | | | | | | | | | | |
| Gialamas et al. 2019 | P | G | F | NA | P | G | NA | G | | Moderate |
| Holmes et al. 2006 | P | F | P | P | P | P | P | P | | Weak |
| Kirk et al. 2014 | P | F | F | P | P | P | F | F | | Weak |
| Kirk and Kirk 2016 | P | F | P | P | P | G | P | P | | Weak |
| Mavilidi et al. 2015 | P | G | P | P | F | G | P | P | | Weak |
| Mavilidi et al. 2016 | P | G | F | P | G | G | F | P | | Weak |
| Mavilidi et al. 2017 | P | G | F | P | G | G | F | P | | Weak |
| Mavilidi et al. 2018 | P | G | G | P | G | G | P | F | | Weak |
| Palmer et al. 2013 | P | F | P | P | F | P | F | F | | Weak |
| Webster et al. 2015 | P | G | F | P | G | F | F | P | | Weak |
| **Cardiovascular Health** | | | | | | | | | | |
| Metcalf et al. 2008 | F | G | G | NA | G | F | NA | F | | High |
| Metcalf et al. 2009 | F | G | P | NA | G | F | NA | F | | High |
| Proudfoot et al. 2019 | F | G | G | NA | F | G | NA | G | | High |
| Leppanen et al. 2017 | P | G | P | NA | F | G | NA | G | | Moderate |
| DuRant et al. 1993 | P | G | P | NA | G | F | NA | P | | Weak |
| Ketelhut et al. 2018 | P | G | G | P | P | F | P | F | | Weak |
| Scheffler et al. 2007 | P | F | P | P | F | P | P | P | | Weak |
| **Social-Emotional Development** | | | | | | | | | | |
| Gialamas et al. 2019 | P | G | F | NA | P | G | NA | G | | Moderate |
| Vella et al. 2015 | P | G | F | NA | F | P | NA | G | | Moderate |
| **Bone Health** | | | | | | | | | | |
| Wosje et al. 2009 | P | G | G | NA | G | G | NA | F | | High |
| Clark et al. 2008 | F | G | P | NA | f | P | NA | G | | Moderate |

^1^ Items D and G were not applicable for longitudinal studies, NA = Not applicable.

**References**

1. Goldfield, G.S., et al., *Effects of Child Care Intervention on Physical Activity and Body Composition.* Am J Prev Med, 2016. **51**(2): p. 225-231.

2. Krombholz, H., *The impact of a 20-month physical activity intervention in child care centers on motor performance and weight in overweight and healthy-weight preschool children.* Perceptual & Motor Skills, 2012. **115**(3): p. 919-932.

3. Mo-suwan, L., et al., *Effects of a controlled trial of a school-based exercise program on the obesity indexes of preschool children.* Am J Clin Nutr, 1998. **68**(5): p. 1006-11.

4. Scheffler, C., K. Ketelhut, and I. Mohasseb, *Does physical education modify the body composition?--results of a longitudinal study of pre-school children.* Anthropol Anz, 2007. **65**(2): p. 193-201.

5. Carter, P.J., et al., *Longitudinal analysis of sleep in relation to BMI and body fat in children: the FLAME study.* Bmj, 2011. **342**: p. d2712.

6. De Coen, V., et al., *Risk factors for childhood overweight: a 30-month longitudinal study of 3- to 6-year-old children.* Public Health Nutr, 2014. **17**(9): p. 1993-2000.

7. Jago, R., et al., *BMI from 3-6 y of age is predicted by TV viewing and physical activity, not diet.* International Journal of Obesity, 2005. **29**(6): p. 557-564.

8. Metcalf, B.S., et al., *Physical activity at the government-recommended level and obesity-related health outcomes: a longitudinal study (Early Bird 37).* Arch Dis Child, 2008. **93**(9): p. 772-7.

9. Wells, J.C. and P. Ritz, *Physical activity at 9-12 months and fatness at 2 years of age.* Am J Hum Biol, 2001. **13**(3): p. 384-9.

10. Saldanha-Gomes, C., et al., *Prospective associations between energy balance-related behaviors at 2 years of age and subsequent adiposity: the EDEN mother-child cohort.* Int J Obes (Lond), 2017. **41**(1): p. 38-45.

11. Butte, N.F., et al., *Role of physical activity and sleep duration in growth and body composition of preschool-aged children.* Obesity (Silver Spring), 2016. **24**(6): p. 1328-35.

12. Ip, E.H., et al., *Physical Activity States of Preschool-Aged Latino Children in Farmworker Families: Predictive Factors and Relationship With BMI Percentile.* Journal of Physical Activity & Health, 2016. **13**(7): p. 726-732.

13. Leppänen, M.H., et al., *Longitudinal Physical Activity, Body Composition, and Physical Fitness in Preschoolers.* Medicine & Science in Sports & Exercise, 2017. **49**(10): p. 2078-2085.

14. Moore, L.L., et al., *Does early physical activity predict body fat change throughout childhood?* Preventive Medicine: An International Journal Devoted to Practice and Theory, 2003. **37**(1): p. 10-17.

15. Donath, L., et al., *Fundamental movement skills in preschoolers: A randomized controlled trial targeting object control proficiency.* Child: Care, Health and Development, 2015. **41**(6): p. 1179-1187.

16. Goodway, J.D. and C.F. Branta, *Influence of a motor skill intervention on fundamental motor skill development of disadvantaged preschool children.* Res Q Exerc Sport, 2003. **74**(1): p. 36-46.

17. Iivonen, S., A. Sääkslahti, and K. Nissinen, *The development of fundamental motor skills of four‐ to five‐year‐old preschool children and the effects of a preschool physical education curriculum.* Early Child Development and Care, 2011. **181**(3): p. 335-343.

18. Ketelhut, K., I. Mohasseb, and R.G. Ketelhut, *Two years of regular exercise decreases blood pressure and improves motor skills in early childhood.* Sport Sciences for Health, 2018. **14**(3): p. 571-578.

19. Lee, H.-M. and J.C. Galloway, *Early Intensive Postural and Movement Training Advances Head Control in Very Young Infants.* Physical Therapy, 2012. **92**(7): p. 935-947.

20. Lobo, M.A. and J.C. Galloway, *Enhanced handling and positioning in early infancy advances development throughout the first year.* Child Development, 2012. **83**(4): p. 1290-1302.

21. Sigmundsson, H. and B. Hopkins, *Baby swimming: exploring the effects of early intervention on subsequent motor abilities.* Child Care Health Dev, 2010. **36**(3): p. 428-30.

22. Venetsanou, F. and A. Kambas, *How can a traditional Greek dances programme affect the motor proficiency of pre‐school children?* Research in Dance Education, 2004. **5**(2): p. 127-138.

23. Kuo, Y.-L., et al., *The influence of wakeful prone positioning on motor development during the early life.* Journal of Developmental and Behavioral Pediatrics, 2008. **29**(5): p. 367-376.

24. Holmes, R.M., A.D. Pellegrini, and S.L. Schmidt, *The effects of different recess timing regimens on preschoolers' classroom attention.* Early Child Development and Care, 2006. **176**(7): p. 735-743.

25. Kirk, S.M., et al., *Using Physical Activity to Teach Academic Content: A Study of the Effects on Literacy in Head Start Preschoolers.* Early Childhood Education Journal, 2014. **42**(3): p. 181-189.

26. Kirk, S.M. and E.P. Kirk, *Sixty Minutes of Physical Activity per Day Included Within Preschool Academic Lessons Improves Early Literacy.* J Sch Health, 2016. **86**(3): p. 155-63.

27. Mavilidi, M.-F., et al., *Effects of Integrated Physical Exercises and Gestures on Preschool Children’s Foreign Language Vocabulary Learning.* Educational Psychology Review, 2015. **27**(3): p. 413-426.

28. Mavilidi, M.-F., et al., *Infusing Physical Activities Into the Classroom: Effects on Preschool Children's Geography Learning.* Mind, Brain, and Education, 2016. **10**(4): p. 256-263.

29. Mavilidi, M.-F., et al., *Effects of Integrating Physical Activities Into a Science Lesson on Preschool Children's Learning and Enjoyment.* Applied Cognitive Psychology, 2017. **31**(3): p. 281-290.

30. Mavilidi, M.-F., et al., *Immediate and delayed effects of integrating physical activity into preschool children’s learning of numeracy skills.* Journal of Experimental Child Psychology, 2018. **166**: p. 502-519.

31. Palmer, K.K., M.W. Miller, and L.E. Robinson, *Acute exercise enhances preschoolers' ability to sustain attention.* J Sport Exerc Psychol, 2013. **35**(4): p. 433-7.

32. Webster, E.K., D.D. Wadsworth, and L.E. Robinson, *Preschoolers' time on-task and physical activity during a classroom activity break.* Pediatr Exerc Sci, 2015. **27**(1): p. 160-7.

33. Metcalf, B.S., et al., *Objectively measured physical activity and its association with adiponectin and other novel metabolic markers: a longitudinal study in children (EarlyBird 38).* Diabetes Care, 2009. **32**(3): p. 468-73.

34. DuRant, R.H., et al., *Association among serum lipid and lipoprotein concentrations and physical activity, physical fitness, and body composition in young children.* J Pediatr, 1993. **123**(2): p. 185-92.

35. Vella, S.A., C.A. Magee, and D.P. Cliff, *Trajectories and predictors of health-related quality of life during childhood.* The Journal of Pediatrics, 2015. **167**(2): p. 422-427.

36. Wosje, K.S., et al., *Adiposity and TV viewing are related to less bone accrual in young children.* J Pediatr, 2009. **154**(1): p. 79-85.e2.

37. Clark, E.M., A.R. Ness, and J.H. Tobias, *Vigorous Physical Activity Increases Fracture Risk in Children Irrespective of Bone Mass: A Prospective Study of the Independent Risk Factors for Fractures in Healthy Children.* Journal of Bone & Mineral Research, 2008. **23**(7): p. 1012-22.
